# Assessing Venous Congestion in Heart Failure: A Review of Splanchnic, Cardiac, and Pulmonary Ultrasound: Part 2: Contrast-Enhanced Ultrasound and Shear Wave

**DOI:** 10.3390/jcm15031111

**Published:** 2026-01-30

**Authors:** Francesco Giangregorio, Esther Centenara, Samanta Mazzocchi, Luigi Gerra, Francesco Tursi, Davide Imberti, Daniela Aschieri

**Affiliations:** 1 Department of Internal Medicine, Castel San Giovanni Hospital, Vle II Giugno, 1, 29015 Castel San Giovanni, Italy; 2 Department of Cardiology, Piacenza Hospital, Via Taverna 49, 29121 Piacenza, Italy; 3 Cardiac and Pneumological Rehabilitation Medicine, Codogno Hospital, 26845 Lodi, Italy; 4 Department of Internal Medicine, Piacenza Hospital, Via Taverna 49, 29121 Piacenza, Italy

**Keywords:** heart failure, congestion, splanchnic circulation, contrast-enhanced ultrasound (CEUS), contrast-enhanced echocardiography, shear wave elastography (SWE), portal vein, hepatic vein, liver stiffness, microbubbles, cardiorenal syndrome, venous excess ultrasound (VExUS)

## Abstract

**Background**: Heart failure (HF) is a systemic syndrome characterized by venous congestion, which critically involves the splanchnic circulation. Conventional assessment methods often lack sensitivity for early or regional congestion. **Methods**: We conducted a systematic review of studies utilizing contrast-enhanced ultrasound (CEUS) and shear wave elastography (SWE) to evaluate congestion in adult HF patients, synthesizing evidence up to July 2025. **Results**: The integrated evidence demonstrates that CEUS and SWE provide distinct, complementary quantitative data. CEUS acts as a functional pillar, detecting microvascular congestion through parameters like prolonged hepatic vein transit time. SWE serves as a structural pillar, quantifying tissue stiffness that correlates with central venous pressure, tracks decongestion, and independently predicts adverse outcomes. Together, they differentiate reversible hemodynamic congestion from irreversible fibrotic remodeling across the liver, spleen, kidneys, and heart. **Conclusions**: Integrating CEUS and SWE into a multi-parametric ultrasound framework provides a comprehensive, bedside assessment of systemic congestion in HF. This approach enhances early detection, improves risk stratification, and offers a potential tool for guiding and monitoring personalized decongestive therapy, representing a significant advancement in holistic HF management.

## 1. Introduction

**Figure 1:**
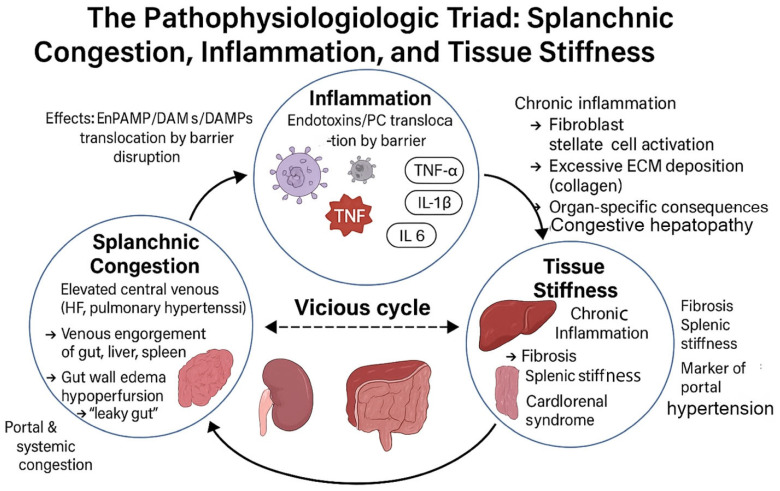
The pathophysiological triad of splanchnic congestion, inflammation, and tissue stiffness.

The splanchnic circulation, a high-compliance, high-capacity vascular bed comprising the gastric, small intestinal, colonic, pancreatic, hepatic, and splenic vasculature, is integral to systemic hemodynamics and metabolic homeostasis. The pathophysiology of heart failure is already explained elsewhere [1]. In the initial phase of heart failure (HF), splanchnic congestion arises from a pathological elevation in venous pressure, most commonly secondary to right-sided heart failure, pulmonary hypertension, or advanced liver cirrhosis. This relationship forms a vicious, self-perpetuating cycle between splanchnic congestion, systemic inflammation, and tissue stiffness, where each component relentlessly fuels the others, creating a downward clinical spiral [2] (Figure 1).

The pathophysiology of advanced heart failure extends far beyond the cardiac pump, initiating a systemic cascade that profoundly impacts abdominal organs. This sequence often begins with splanchnic congestion, a direct consequence of elevated central venous pressure secondary to right heart dysfunction [3]. This retrograde transmission of pressure engorges the venous networks of the gut, liver, spleen, and kidneys, creating a state of systemic venous hypertension characterized by capillary engorgement and interstitial edema [4,5]. The initial site of this cascade, the gastrointestinal tract, suffers critical injury. Gut wall edema and hypoperfusion disrupt the integrity of the intestinal mucosal barrier, leading to a state of increased permeability often termed a ‘leaky gut’ [6,7].

In liver, Hepatic congestion in heart failure results from elevated central venous pressure transmitted backward through the hepatic veins, leading to passive liver congestion. This engorgement causes sinusoidal dilation, hepatic hypoperfusion, and centrilobular hepatocyte necrosis. Over time, this process can progress to congestive hepatopathy and cardiac cirrhosis, characterized by fibrosis and impaired synthetic function. The resulting liver dysfunction manifests through elevated transaminases, coagulopathy, and jaundice, indicating a poor prognostic sign in advanced heart failure [8]. Concurrently, splenic congestion manifests as splenomegaly and it is a hallmark of portal hypertension, resulting from increased resistance in the portosystemic circulation [9]. This state of persistent congestion is a potent driver of localized inflammation. Splenic congestion can promote the retention and lysis of red blood cells, leading to iron deposition and the activation of immune cells, which amplifies a local and systemic inflammatory response [10]. This inflammation is increasingly recognized as a key mediator of disease progression in both cardiac and hepatic pathologies. In the kidney, this renal venous hypertension compromises the delicate pressure gradient essential for glomerular filtration, directly contributing to the pathophysiology of cardiorenal syndrome [11].

This breach allows for the translocation of bacterial endotoxins, such as lipopolysaccharide (LPS), into the portal circulation—a process known as bacterial translocation [12].

Conditions like heart failure, initiates damaging inflammatory processes in two key organs: the kidneys and the gastrointestinal tract. The central discursive theme is that congestion begets inflammation, which in turn perpetuates organ damage and instability.

The process begins in the congested kidney. Here, edema and reduced blood flow create a state of hypoxia in the tubulointerstitial space. This oxygen deficiency acts as a trigger, activating local fibroblasts and drawing in pro-inflammatory macrophages [13]. These cells release a flood of cytokines, such as TNF-α, IL-6, and the potent pro-fibrotic agent TGF-β1, while also promoting oxidative stress. This combination directly injures tubular cells and, crucially, sets in motion fibrotic pathways, laying the groundwork for permanent structural damage and functional decline [13].

Simultaneously, a parallel and interconnected drama unfolds in the congested gut. The text establishes a “well-established nexus” between congestion and systemic inflammation [14]. Venous hypertension causes bowel wall edema, which compromises the integrity of the intestinal mucosal barrier. This breach allows gut-derived bacterial products, known as pathogen-associated molecular patterns (PAMPs) like lipopolysaccharide (LPS), to translocate into the portal and systemic circulation [15]. This event is termed bacterial translocation and acts as a potent “ignition switch” for a widespread immune response [14].

The ensuing humoral and innate inflammatory response is robust. The translocated PAMPs bind to Toll-like receptors (TLRs) on immune cells, sparking a cascade that produces a storm of pro-inflammatory cytokines, including TNF-α, IL-1β, and IL-6 [16]. This creates a self-perpetuating systemic inflammatory state. The text concludes by noting that this inflammation not only causes further damage to end-organs (like the already struggling kidneys) but also contributes to worsening hemodynamic instability, potentially creating a vicious cycle that exacerbates the original congestion [16].

Thus, a localized hemodynamic problem rapidly ignites a systemic inflammatory response. This systemic inflammation is mediated through both humoral and innate immune pathways. The influx of pathogen-associated molecular patterns (PAMPs) activates immune cells via Toll-like receptors (TLRs), particularly TLR4, triggering a robust release of pro-inflammatory cytokines, including TNF-α, IL-1β, and IL-6 [16,17,18]. This cytokine storm not only perpetuates end-organ damage but also contributes to further hemodynamic instability and cardiac remodeling, with TLR4 implicated in driving pathological hypertrophy and fibrosis within the heart itself [19,20].

A critical cellular orchestrator in the transition from congestion to inflammation is the mast cell (MC). Activated by the mechanical stress of elevated venous pressure (mechanotransduction) and metabolic signals, mast cells in the splanchnic bed degranulate, releasing mediators like TNF-α, VEGF, histamine, and proteases [21]. These compounds drive local pathology—increasing vascular permeability, promoting edema and angiogenesis—and have systemic cardiovascular consequences, such as exacerbating cardiac preload [21,22]. This mechanism is directly relevant to heart failure, where systemic congestion likely activates mast cells through similar pathways, contributing to congestive enteropathy [23]. Within the failing heart, increased mast cell density and their mediators (e.g., chymase) further promote inflammation, fibrosis, and adverse remodeling [23,24,25]. Notably, mast cell populations are heterogeneous, with both pro-fibrotic (CMC2) and potentially reparative (CMC1) subtypes influencing disease progression [26], making them a target for emerging therapies [27,28,29,30,31,32]. The final, detrimental outcome of this sustained congestion and inflammation is progressive tissue fibrosis and stiffening. Across organs, persistent inflammatory signaling and mechanical stress activate fibroblast populations: In the liver, hepatic stellate cells are activated, leading to excessive collagen deposition and the development of congestive hepatopathy, which can progress to cardiac cirrhosis [33,34]. In the kidney, hypoxia and cytokines activate myofibroblasts, resulting in tubulointerstitial fibrosis and a loss of renal compliance [13,35]. In the spleen, chronic congestion drives collagen deposition, increasing splenic stiffness, which correlates with the severity of portal hypertension [36]. This progression from congestion to inflammation and, ultimately, to fibrosis establishes a vicious, self-perpetuating cycle that significantly worsens clinical outcomes.

Advanced Ultrasound: A Window into the Cascade: Modern ultrasound techniques provide a unique, non-invasive means to visualize and quantify these distinct yet concurrent pathophysiological processes. Contrast-enhanced ultrasound (CEUS) offers a dynamic, functional assessment of hemodynamics and microvascular integrity. By tracking intravascular microbubbles, CEUS can quantify organ-specific perfusion kinetics [37]. Additionally, the availability of ultrasound contrast agents and their approved indications vary globally, creating disparities in access and standardized use. Despite these constraints, when applied within its validated scope—such as in characterizing indeterminate lesions, guiding interventions, or monitoring ablation sites—CEUS proves to be an invaluable, safe, and efficient modality that enhances diagnostic confidence and can streamline patient management pathways [38].

In congestion, impaired venous outflow manifests as a prolongation of transit times, such as delayed hepatic vein arrival [39]. Conversely, inflammatory processes, with their associated angiogenesis and increased vascular permeability, can be detected through patterns of progressive, heterogeneous enhancement as microbubbles pool within the tissue [40,41]. Thus, CEUS can reveal the early hemodynamic dysfunction of congestion and the subsequent inflammatory response in abdominal, cardiac, and thoracic sites [42,43,44], with techniques like dynamic contrast-enhanced (DCE-US) allowing for perfusion quantification [45]. Shear wave elastography (SWE) complements CEUS by providing a structural assessment of tissue stiffness [46] and degree of steatosis [47]. It quantifies the speed of shear waves propagated through tissue, which increases with higher stiffness [48]. 

This allows SWE to directly measure the mechanical consequences of the pathophysiological triad:

- Acutely, elevated stiffness primarily reflects interstitial edema and pressure from congestion [49].
- Chronically, persistent stiffness indicates irreversible fibrotic remodeling [46,49].

SWE has been validated for staging fibrosis in the liver [46] and is increasingly applied to assess stiffening in the kidney [50], spleen [51], and lungs. A critical limitation is that conditions like active inflammation or severe steatosis can acutely elevate stiffness measurements independent of fibrosis, requiring careful interpretation [46].

In conclusion, heart failure triggers a deleterious systemic cascade: splanchnic venous congestion leads to barrier dysfunction and bacterial translocation, which fuels a systemic inflammatory response orchestrated in part by mast cells. This inflammatory milieu, combined with persistent mechanical stress, drives progressive fibrosis and organ stiffening. The integrated use of CEUS and SWE allows clinicians to move beyond anatomy, functionally characterizing congestion and inflammation while structurally quantifying the resultant tissue remodeling. This non-invasive window into the cardiorenal-hepatic and cardio-splenic axes is pivotal for distinguishing reversible hemodynamic stress from irreversible fibrotic damage, thereby guiding therapy, prognostic stratification, and the development of targeted interventions aimed at breaking this vicious cycle.

This review aims to systematically examine and synthesize current evidence on the use of advanced ultrasonography—specifically contrast-enhanced ultraSound (CEUS) and shear wave elastography (SWE)—for the evaluation of splanchnic, thoracic, and cardiac alterations in patients with heart failure (HF). By integrating data from existing studies, this review intends to identify consistent contrastographic or elsatosonographic patterns that correlate with hemodynamic status and adverse clinical outcomes. The central hypothesis underpinning this review is that the ultrasonographic evaluation of the abdominal, cardiac, and thoracic districts yields clinically meaningful insights into the pathophysiology and severity of venous congestion in heart failure. It is proposed that these sonographic parameters not only reflect systemic hemodynamic burden but may also serve as valuable prognostic markers and therapeutic guides. Furthermore, the review postulates that integrating advanced ultrasound findings could enhance early detection, improve risk stratification, and optimize decongestive management in both acute and chronic heart failure.

## 2. Materials and Methods

### 2.1. Search Strategy

A comprehensive and systematic search in PubMed was conducted to identify relevant literature, utilizing a preplanned, reproducible search strategy. F.G F.G. designed the study, performed the search, and analyzed the data. F.G., F.T., D.I., and D.A. reviewed and corrected the manuscript. The search terms used were: (“Contrast-enhanced Ultrasound” OR “CEUS” OR “elastography” OR “shear wave” OR “stiffness”) combined with (“Heart Failure” [Mesh] OR “cardiac failure” OR “ventricular dysfunction” OR “HFpEF” OR “HFrEF”). We applied no restrictions regarding the date of publication, covering all articles published up to July 2025.

### 2.2. Study Selection

We systematically screened titles, abstracts, and full texts to determine their eligibility according to the Prisma 2020 statement [52] (Table A1 in Appendix A). FINQUI The inclusion criteria were as follows: (1) language: articles published in English, Spanish, or Italian; (2) type of study: experimental, observational, and systematic review articles, published as original research in peer-reviewed journals, and limited to human studies; (3) population: adult patients diagnosed with any type of heart failure; (4) focus: studies examining changes in splanchnic vascularization and their impact on heart failure progression or management; and (5) outcomes: studies that measured physiological parameters of splanchnic circulation and related these to heart failure outcomes. The exclusion criteria included the following: (1) case reports, opinion papers, editorials, and studies available only as abstracts; (2) pediatric studies; (3) studies focusing on non-heart failure populations; and (4) studies utilizing non-standard methods of assessing splanchnic vascularization.

### 2.3. Data Extraction

Following initial screening, selected articles underwent a detailed review. Data extracted included the following: first author, year of publication, country of the study, sample size, details of the splanchnic vascular assessment techniques used (e.g., ultrasound, MRI), specific parameters measured (e.g., blood flow velocity, vascular resistance), and key findings relevant to heart failure management and outcomes. A total of 320 records were identified through electronic databases, including Medline (n = 260), EMBASE (n = 60), and the Cochrane Central Register of Controlled Trials (n = 0). Additional records were retrieved from supplementary sources, comprising zero from websites and five from citation searching, while no records were obtained from organizational repositories. After removal of 4 duplicates and 159 records excluded for other reasons, 157 unique records remained for screening. Title and abstract screening excluded 23 records, leaving 17 reports sought for retrieval. Of these, 28 could not be retrieved, resulting in 106 full-text reports assessed for eligibility.

**Figure 2:**
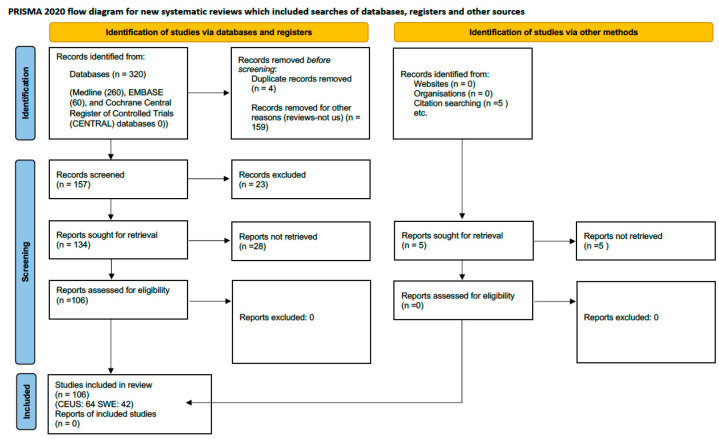
Flow chart of the literature search according to the Prisma 2020 statement [52].

Ultimately, 106 studies fulfilled the inclusion criteria and were incorporated into the review. The selection process is detailed in the PRISMA 2020 flow diagram (Figure 2). Reviewer disagreements regarding study eligibility and data interpretation were resolved through discussion and consensus with a third independent reviewer to ensure objectivity and methodological consistency. Reference management was performed using EndNote 21, version 21.5 (Clarivate Analytics), running under the macOS 15.27 operating system, which facilitated accurate data management and duplication control.

#### Risk of Bias and Certainty Assessment

The pronounced heterogeneity among the included studies—spanning design, imaging protocols, and measured outcomes—precluded a meaningful quantitative synthesis and the application of standardized risk-of-bias tools (e.g., Newcastle–Ottawa Scale [53], ROB 2.0 [54]). These tools are most effective when comparing methodologically similar studies. Our approach instead prioritized a rigorous qualitative synthesis. We systematically identified and narratively described key methodological limitations (such as sample size, blinding, and potential for confounding) as they pertained to the findings of individual studies and the overall body of evidence. This review acknowledges the lack of a prospectively registered protocol as a limitation, a measure that guards against bias in hypothesis-driven analyses. However, as a broad, exploratory synthesis of a heterogeneous field, its narrative approach was inherently iterative, making a rigid, pre-specified protocol less practical. The risk of selective reporting was lower given the aim to map all evidence, not test a single hypothesis. To ensure rigor, we adhered strictly to PRISMA 2020 guidelines, with an internally defined, pre-planned methodology and full transparency, providing a verifiable audit trail. This foundational overview paves the way for future, protocol-driven reviews as the field matures.

### 2.4. Data Synthesis and Analysis Framework

Given the pronounced methodological and clinical heterogeneity among the included studies—spanning diverse designs (e.g., prospective cohorts, case–control, cross-sectional), patient populations (acute vs. chronic HF, varying HF phenotypes), ultrasound acquisition protocols, and reported outcomes—a formal meta-analysis was deemed inappropriate. Instead, a systematic narrative synthesis was conducted, adhering to established guidelines for the synthesis of heterogeneous evidence.

To ensure a coherent synthesis of the heterogeneous evidence, our analytical framework was explicitly designed to differentiate between two fundamental pathophysiological states:

I. Hemodynamic (or Acute) Congestion: Characterized by reversible increases in venous pressure and interstitial fluid, typically reflected in prolonged transit times on CEUS (e.g., hepatic vein transit time) and acute, potentially reversible elevations in tissue stiffness on SWE.
II. Fibrotic (or Chronic) Remodeling: Characterized by irreversible structural change due to extracellular matrix deposition, primarily quantified by persistently elevated tissue stiffness on SWE that does not normalize with decongestive therapy.

This distinction is central to the review’s hypothesis. By mapping study outcomes onto this dichotomy, the synthesis evaluates the ability of CEUS and SWE not only to detect congestion but, more importantly, to discriminate its reversible hemodynamic component from the progressive, irreversible fibrotic organ damage it may incite. This directly informs prognosis and therapeutic strategy.

We developed a structured analytic framework grounded in the physiological domains most relevant to congestion assessment—namely, microvascular perfusion (contrast-enhanced ultrasound), tissue biomechanics (shear wave elastography), and conventional macrocirculatory ultrasound approaches. This framework enabled standardized mapping of heterogeneous outcomes onto a unified interpretative model. The synthesis prioritized clarity in distinguishing ultrasonographic metrics that reflect hemodynamic congestion (transit times, venous flow patterns) from those that indicate structural organ sequelae (tissue stiffness, chronic remodeling).

Data extracted from eligible studies in the attached dataset were organized according to the following thematic pillars:

(1) CEUS-Derived Microvascular Perfusion Metrics:

This included hepatic venous transit time (HVTT), hepatic artery–hepatic vein transit time (HA–HVTT), splenic and renal enhancement parameters, and the quantification of perfusion defects or delayed wash-in/wash-out kinetics. Studies were synthesized by (a) methodological approach (agent, dose, ROI selection), (b) pathophysiological target (congestion vs. inflammation vs. hypoperfusion), and (c) correlation with clinical or invasive hemodynamic markers.

(2) Elastography-Derived Tissue Stiffness Parameters:

Findings related to liver stiffness measurement (LSM), spleen stiffness, myocardial stiffness, and renal cortical stiffness were collated with attention to the distinction between reversible stiffness elevations attributable to congestion and irreversible elevations linked to fibrosis. Studies employing both 2D-SWE and pSWE were analyzed for comparability across technical platforms and diagnostic thresholds.

(3) Integrated Multi-Organ Ultrasound Protocols:

Where studies combined CEUS, SWE, and conventional Doppler indices, the synthesis focused on evaluating the additive diagnostic value of multimodal imaging and the ability of these combined approaches to discriminate acute from chronic organ injury. Particular attention was afforded to workflows echoing the conceptual structure of the diagnostic diagram provided in the source document (Figure 1), emphasizing the “Two-Pillar Model” (functional perfusion vs. structural stiffness) as a recurring organizing theme.

(4) Clinical and Prognostic Outcomes:

Outcomes, such as HF hospitalization, mortality, renal deterioration, and response to decongestion, were aggregated and compared across imaging modalities. Because the included studies differed widely in sample size, disease severity, imaging endpoints, and follow-up duration, conclusions were drawn using graded confidence (strong, moderate, weak) based on the consistency and volume of supporting evidence.

In line with the methodological strategy of conventional ultrasound review [1], the synthesis describes points of convergence across studies, recurrent pathophysiological signatures, and areas of discordance. When applicable, findings were contextualized relative to invasive hemodynamic data, biochemical congestion markers, and established clinical endpoints. Deviations in methodology (e.g., contrast agent type, probe frequency, timing of CEUS acquisition, or SWE operator dependence) were explicitly noted when they influenced interpretation.

Importantly, contradictory or inconclusive findings were not excluded but were instead integrated into the synthesis to highlight unresolved questions and areas requiring further investigation. This includes, for example, variations in SWE cut-off values for differentiating congestion from fibrosis, or inconsistencies in CEUS perfusion metrics obtained in heterogeneous renal phenotypes.

This framework ultimately enabled a systematic comparison of conventional and advanced ultrasound techniques, allowing the Section 3 to provide a coherent, physiologically anchored narrative despite the heterogeneity of available data.

## 3. Results

Building upon the foundation of conventional ultrasound, advanced techniques such as CEUS and SWE have emerged, capable of providing a sophisticated, real-time analysis of tissue characteristics.

ADVANCED ULTRASONOGRAPHIC TECHNIQUES: CEUS AND SWE

**Contrast-enhanced ultrasound:** Contrast-enhanced ultrasound (CEUS) is an advanced imaging technique that expands conventional sonography by using intravenous ultrasound contrast agents (UCAs), which are typically micron-sized, gas-filled microbubbles stabilized by a shell [55]. Unlike other contrast agents, these microbubbles are true blood-pool agents, remaining within the vascular compartment [55]. Unlike CT or MRI agents, ultrasound microbubbles remain intravascular [55]. Their diagnostic power stems from nonlinear oscillation under ultrasound, producing unique harmonic echoes distinct from tissue [39]. Modern ultrasound systems use low-mechanical-index software to selectively detect microbubble signals, suppressing tissue background for real-time blood-flow images [39]. This visualizes microvascular perfusion. While primarily for characterizing focal liver lesions via vascular patterns, CEUS applications now include renal, pancreatic, and cardiovascular imaging [56]. A key CEUS advantage is continuous, high-resolution vascular assessment without ionizing radiation and with a strong safety profile. This is enabled by ultrasound contrast agents (UCAs)—gas microbubbles that circulate like red blood cells to enhance the ultrasound signal [57].

As blood-pool tracers, ultrasound contrast agents circulate intravascularly, enabling real-time imaging of macro- and microvascular flow [57]. Contrast-specific techniques separate microbubble signals from tissue. Analyzing the dynamic wash-in and wash-out of the bolus allows quantitative evaluation of tissue perfusion and vascular architecture [37]. This repeatable, bedside-capable method is valuable for guiding interventions and monitoring therapy [37].

Ultrasound contrast agents dissolve quickly. The first, Levovist^TM^ (Bayer AG), used galactose crystals [58]. Key modern agents are perfluoro gas-based, like SonoVue^TM^ (Bracco Imaging, Milan, Italy) and Definity^TM^ (Lantheus Medical Imaging, Bedford, MA, USA), which use phospholipid membranes [58]. A newer agent, Sonazoid^®^ (perfluorobutane microspheres), was presented in 2008. In 2008, a new contrast agent, perfluorobutane (PFB) microspheres (Sonazoid^TM^, GE Healthcare, Oslo, Norway) was presented [59]. It consists of perfluorobutane (PFB) microspheres stabilized by a monomolecular membrane of hydrogenated egg phosphatidylserine, embedded in an amorphous sucrose structure. It maintains good stability after reconstitution, showing no significant changes in physicochemical properties for at least 2 h. PFB enables diagnosis of focal liver lesions in vascular-phase imaging and enhanced detection of lesions in Kupffer-phase imaging [60].

**Figure 3:**
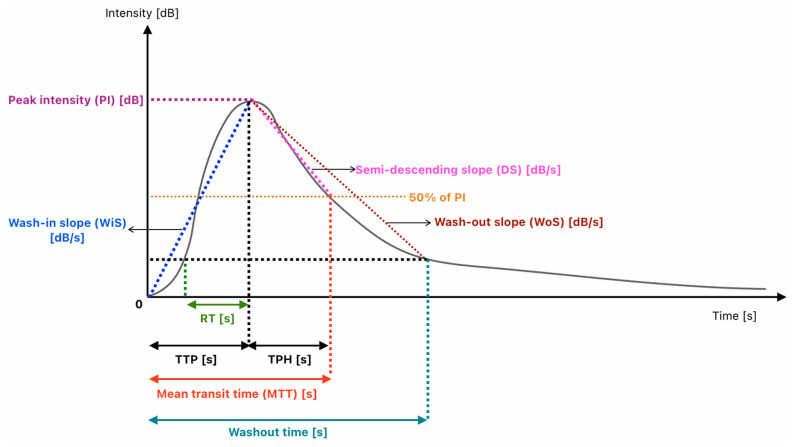
Time–intensity curves: A time–intensity curve (TIC) provides a graphical representation of the kinetic behavior of an ultrasound contrast agent within a selected tissue region. The analysis begins with a baseline, the flat signal before contrast arrival, with time zero marking the injection. The wash-in phase follows, depicted by the curve’s ascent as the contrast agent enters the microvasculature. This rise is characterized by the rise time (RT), the delay until the initial increase, and the wash-in slope (WIS), whose steepness reflects the rate of inflow and blood flow velocity. The curve culminates at peak enhancement, defined by the peak intensity (PI), a measure proportional to local blood volume, and the time-to-peak (TTP), the duration to reach this maximum. Subsequently, the wash-out phase shows the signal decline as contrast clears. This descent is quantified by the time from peak to one-half signal intensity (TPH), the descending slope (DS) indicating clearance rate, and the total wash-out time. A key integrative parameter is the mean transit time (MTT), representing the average time contrast spends within the vasculature. Together, these elements transform the TIC into a quantitative map of tissue perfusion dynamics.

CEUS is a powerful, safe, and versatile real-time perfusion imaging tool with applications across numerous organs [42]. Its quantitative extension, dynamic contrast-enhanced ultrasound (DCE-US), assesses microvascular perfusion by analyzing time–intensity curves (TICs) depicting ultrasound contrast agent kinetics over time (Figure 3).

The time–intensity curve (TIC) analysis in DCE-US quantifies the wash-in (contrast influx) and wash-out (clearance) phases of pure blood-pool agents. This provides objective data on vascular architecture and flow dynamics, crucial for oncology, moving beyond subjective assessment [61]. The wash-in phase reflects microbubble arrival and accumulation. Key quantitative parameters include rise time (RT) to initial signal increase, time-to-peak (TTP) to maximum peak intensity (PI), and the wash-in rate (WIR) slope, indicating inflow rapidity and influenced by neovascular density and permeability [62]. In tumors, malignant angiogenesis often causes a rapid, intense wash-in, with a steeper slope and higher peak intensity (PI) than benign tissue [62]. The subsequent wash-out phase is characterized by parameters like wash-out time (WOT) and descending slope (DS) [63]. Malignant lesions frequently show rapid wash-out due to disorganized vasculature, unlike the prolonged wash-out of benign lesions. Quantification uses models where signal intensity is proportional to microbubble concentration. To ensure reproducibility, normalization—comparing parameters like tumor PI to those in adjacent normal tissue—is recommended, minimizing technical variability. This allows DCE-US TIC analysis to provide valuable functional characterization of focal lesions for differential diagnosis [64]. More significantly, it enables early assessment of treatment response, especially to anti-angiogenic therapies [63,65,66], by detecting changes in perfusion that precede morphological tumor shrinkage. This addresses a major limitation of traditional size-based criteria like RECIST. Studies have demonstrated that a decrease in AUC or PI, or an increase in MTT, can indicate effective vascular shutdown weeks before tumor volume changes [63,65,66] (Figure 3).

**Shear wave elastography:** SWE represents a significant evolution in medical imaging, moving beyond anatomical depiction to provide a quantitative assessment of tissue mechanical properties. The fundamental premise of this ultrasound-based technique is the application of Hooke’s Law to biological tissues, operating on the principle that stiffer tissues propagate waves at higher velocities than softer, more pliable ones [67,68]. Unlike its predecessor, strain elastography, which required an external compression force from the operator and yielded only a relative, qualitative measure of stiffness, SWE is an active technique that generates its own metric through a precisely controlled internal push (Table A2 in Appendix A).

The process is initiated when the ultrasound transducer delivers a focused acoustic “push” pulse to a specific depth within the tissue of interest. This targeted energy delivery induces a transient, micron-scale displacement, which in turn generates low-frequency shear waves that radiate outward perpendicular to the initial push beam [69]. The genius of the method lies in its subsequent step: the same transducer rapidly switches to a high-frame-rate imaging mode, often termed “ultrafast” imaging, to track the propagation of these shear waves as they move through the surrounding tissue. By measuring the time it takes for the shear wave to reach multiple adjacent points, the system can directly calculate its velocity in meters per second.

This measured shear wave velocity is then automatically converted by the machine’s software into a quantitative value of tissue stiffness, most commonly presented in kilopascals (kPa), providing an absolute and reproducible Young’s modulus value [70]. Consequently, SWE translates the subjective clinical act of palpation into an objective, color-coded map that is superimposed on the conventional B-mode image, allowing radiologists to visually and numerically discriminate between normal and pathologically stiffened tissues. Its most established clinical role is in the non-invasive assessment of liver fibrosis, where it can stage scarring without a biopsy, but its utility is continually expanding into evaluating masses in the breast, thyroid, and prostate, offering a functional complement to standard anatomical scans (Table A2 in Appendix A).

**Figure 4:**
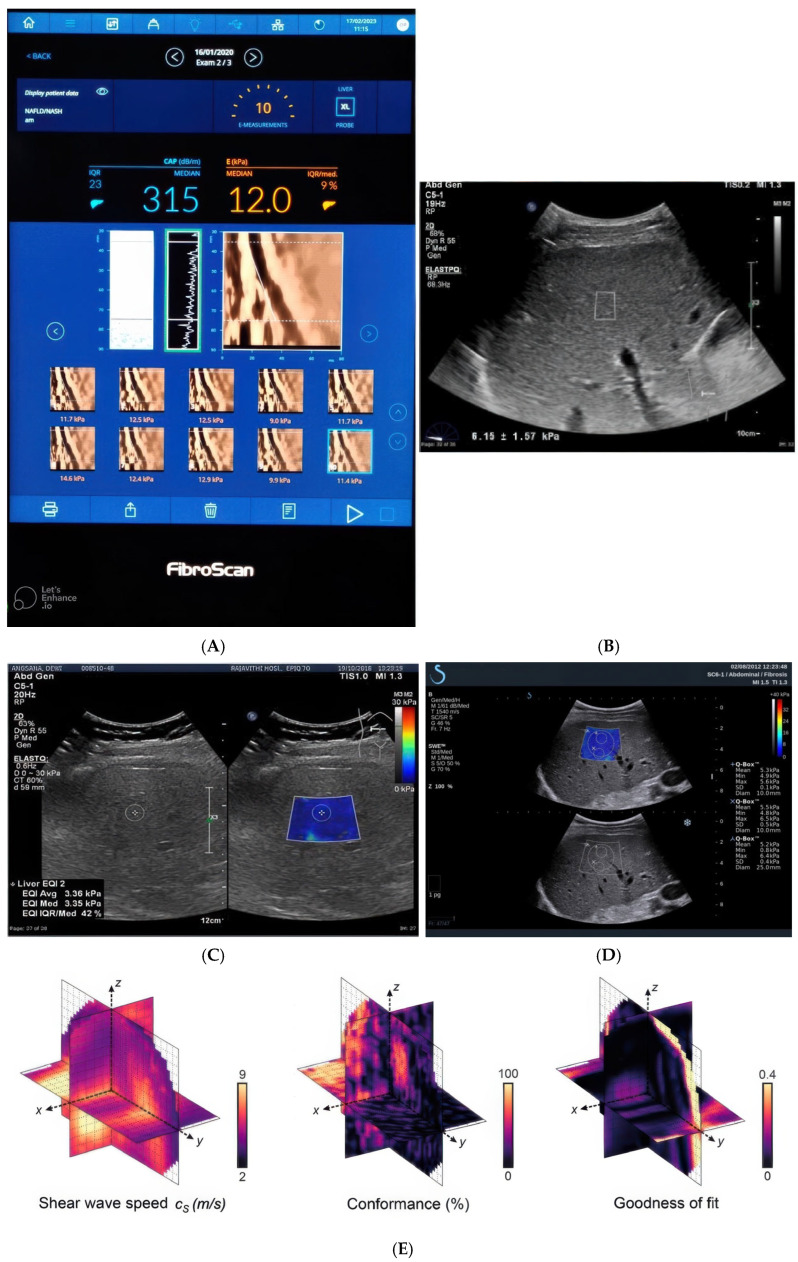
Shear wave typology: (**A**) The technology has evolved into several key implementations: transient elastography (TE): it uses an external mechanical vibrator. It is simple, fast, and the established method for liver fibrosis staging (e.g., FibroScan^®^) [71]. (**B**) Point SWE (p-SWE): it uses acoustic radiation force impulse (ARFI) to assess stiffness at a single point, offering quantitative measurements with standard ultrasound probes [72]. (**C**) 2D-SWE: Rapidly excites multiple focal points to create a real-time, color-coded stiffness map (elastogram) superimposed on a B-mode image, widely used for liver, breast, and thyroid assessment [73]. (**D**) Supersonic Shear Imaging (SSI) [74]: A specific 2D-SWE method that uses a supersonic moving source to generate strong planar shear waves for very fast acquisition. (**E**) 3D-SWE: Extends the concept to provide volumetric stiffness maps, improving spatial assessment of lesions.

The field of medical elastography has diversified into a suite of non-invasive techniques for quantifying tissue stiffness, a key biomarker for pathology (Figure 4). These methods represent a significant evolution from qualitative palpation to objective, image-based biomechanical assessment.

The foundational clinical implementation is transient elastography (TE), such as FibroScan^®^. This technique employs an external mechanical vibrator to generate shear waves, measuring their propagation speed to deduce stiffness. Its strength lies in its simplicity, speed, and robust validation, making it the established first-line tool for staging liver fibrosis [71]. Advancements integrated directly into conventional ultrasound systems led to acoustic radiation force impulse (ARFI)-based techniques. Here, the ultrasound probe itself generates the force. The initial implementation, point shear wave elastography (p-SWE), uses a focused acoustic “push” beam to assess stiffness at a single, user-defined location, providing a quantitative value (e.g., in kPa or m/s) with high reproducibility [72]. Expanding from a single point, 2D shear wave elastography (2D-SWE) represents a major leap. It rapidly excites multiple focal points or a “push” beam to generate a broader field of shear waves. This allows for the real-time creation of a color-coded elastogram (stiffness map), which is superimposed on a standard B-mode anatomical image. This visual and quantitative mapping is invaluable for targeting specific lesions and assessing heterogeneity in organs like the liver, breast, and thyroid. A notable technical refinement within 2D-SWE is Supersonic Shear Imaging (SSI), which uses a rapidly moving acoustic source to create a Mach cone effect, generating strong, planar shear waves for particularly fast and robust elastogram acquisition. The most spatially comprehensive evolution is 3D-SWE, which extends the principle to acquire a volumetric stiffness dataset. By providing a three-dimensional stiffness map of a region of interest, it improves the spatial assessment of lesion boundaries and internal consistency, reducing sampling error and offering a more complete biomechanical profile.

In summary, the trajectory of elastography technology has progressed from a single-point, external mechanical measurement (TE) to integrated, acoustic radiation force-based point sampling (p-SWE), and onward to real-time 2D mapping (2D-SWE/SSI) and ultimately volumetric 3D assessment (3D-SWE). Each implementation offers a different balance of speed, spatial resolution, and quantitative rigor, but all serve the core scientific objective of translating tissue mechanical properties into a clinically actionable diagnostic metric.

SWE has emerged as a pivotal non-invasive tool for assessing liver fibrosis, a key prognostic factor in chronic liver diseases such as non-alcoholic fatty liver disease (NAFLD) and non-alcoholic steatohepatitis (NASH). These technologies quantify tissue stiffness by measuring the velocity of shear waves generated within the liver parenchyma, which correlates with the degree of fibrosis. The main SWE techniques include vibration-controlled transient elastography (VCTE), point shear wave elastography (pSWE), and two-dimensional shear wave elastography (2D-SWE). Each offers distinct technical and clinical profiles [75].

Vibration-controlled transient elastography (VCTE), implemented via the FibroScan^®^ device, was the first widely validated method. It uses a mechanical piston to generate a low-frequency shear wave and measures liver stiffness (LS) in kilopascals (kPa). VCTE is highly standardized and has extensive outcome validation; for example, it shows a summary area under the curve (sAUC) of 0.85 for diagnosing advanced fibrosis (≥F3) in NAFLD [75]. Its main advantages are its robustness and the simultaneous assessment of steatosis via the Controlled Attenuation Parameter (CAP). Limitations include potential inaccuracy in obese patients (though addressed by an XL probe) and the inability to perform B-mode imaging during the exam.

Shear wave elastography (SWE) is a quantitative ultrasound-based imaging technique that measures tissue stiffness by applying the principle of Hooke’s Law, where stiffer tissues propagate shear waves faster than softer ones [67,68]. It represents a major advancement from qualitative strain elastography, as it actively generates its own “push” pulse to create shear waves [69] and provides an absolute, reproducible measure of stiffness in kilopascals (kPa) or meters per second (m/s) [70].

Technical Process: The procedure involves an ultrasound transducer delivering a focused acoustic “push” pulse to induce micron-scale displacement, generating low-frequency shear waves [69]. The same transducer then uses ultrafast imaging to track the propagation speed of these waves. The wave velocity is automatically converted into a quantitative stiffness value (Young’s modulus), which is displayed as a color-coded map over a standard B-mode image [70].

Evolution and Types of Elastography: The field has diversified into several key techniques (Figure 4):

*Transient Elastography (TE)*: Uses an external mechanical vibrator (e.g., FibroScan^®^) and is a validated, first-line tool for liver fibrosis staging [71].

*Point Shear Wave Elastography (p-SWE)*: Uses acoustic radiation force impulse (ARFI) from the ultrasound probe to measure stiffness at a single, user-defined point, offering quantitative values with high reproducibility [72].

*2D Shear Wave Elastography (2D-SWE)*: Generates a real time, color-coded elastogram (stiffness map) over a B-mode image, allowing assessment of tissue heterogeneity. A refinement is Supersonic Shear Imaging (SSI), which uses a rapidly moving acoustic source for fast acquisition [73].

*3D Shear Wave Elastography (3D-SWE)*: Provides volumetric stiffness maps for improved spatial assessment of lesions.

Primary Clinical Application in Liver Fibrosis: SWE is a pivotal non-invasive tool for staging liver fibrosis in conditions like NAFLD and NASH. The main techniques are as follows:

Vibration-controlled transient elastography (VCTE/FibroScan^®^): The first widely validated method. It is highly standardized and can simultaneously assess steatosis via CAP. Its diagnostic accuracy for advanced fibrosis is high (sAUC 0.85), though it can be limited in obese patients and lacks concurrent B-mode imaging [75].

Point SWE (pSWE): Integrated into conventional ultrasound systems, it provides a single stiffness value with B-mode guidance. Its diagnostic performance is comparable to VCTE, and it shows high sensitivity (94%) for detecting varices needing treatment in cirrhosis [76,77].

2D-SWE: Provides a real-time elastogram for improved sampling. It shows excellent diagnostic accuracy (AUCs > 0.88 for advanced fibrosis), and newer systems often combine it with multiparametric assessments like attenuation imaging for steatosis [75,77].

In conclusion, SWE translates the clinical act of palpation into an objective, quantitative metric. While VCTE remains the primary screening tool, pSWE and 2D-SWE offer greater versatility by combining elastography with anatomical imaging. The technology’s trajectory has progressed from single-point measurements (TE) to integrated point sampling (p-SWE), real-time 2D mapping (2D-SWE), and volumetric 3D assessment (3D-SWE). Furthermore, SWE is functionally complementary to contrast-enhanced ultrasound (CEUS); while CEUS assesses microvascularization [39,56], SWE quantifies tissue stiffness [69,78], together providing a comprehensive diagnostic profile of lesions.

Elastography is founded on the biomechanical principle that liver fibrosis increases tissue stiffness, which can be quantified by measuring the velocity of mechanically induced shear waves propagating through the liver parenchyma. These waves are generated either by external vibration in vibration-controlled transient elastography (VCTE) or by acoustic radiation force impulses produced by the ultrasound transducer in ARFI-based techniques, including point and two-dimensional shear wave elastography. The resulting liver stiffness measurements (LSM), expressed in kilopascals or meters per second, correlate with fibrosis severity when acquired using standardized protocols that ensure reliability and reproducibility, including fasting conditions and quality metrics such as the interquartile range/median ratio [79,80].

Clinically, elastography is primarily indicated for non-invasive staging of liver fibrosis in a wide range of chronic liver diseases, including metabolic dysfunction-associated steatotic liver disease, alcohol-related liver disease, and chronic hepatitis B and C [46]. Beyond fibrosis staging, LSM has strong prognostic utility. It enables stratification of patients according to their risk of liver-related events, such as hepatic decompensation, hepatocellular carcinoma, and mortality, with validated stiffness thresholds identifying low- and high-risk populations [81]. Additionally, elastography plays a key role in assessing clinically significant portal hypertension. When combined with platelet count, LSM is incorporated into consensus criteria to safely rule out esophageal varices and reduce the need for screening endoscopy [82]. The advantages of elastography are substantial. It provides a rapid, point-of-care, and repeatable alternative to liver biopsy, minimizing patient risk while maintaining excellent diagnostic accuracy for advanced fibrosis and cirrhosis, with reported areas under the curve exceeding 0.85 across etiologies [83]. Its safety profile, absence of ionizing radiation or nephrotoxicity, applicability in pediatric populations, and cost-effectiveness compared with MRI-based elastography further support its widespread clinical adoption [46,79] (Table A2).

### 3.1. Congestion, Inflammation, and Fibrosis in Splanchnic Organs

We evaluated the international literature about CEUS and SWE of splanchnic organs (liver, spleen, kidney), of the heart, and of the lungs in acute and chronic heart failure:

#### 3.1.1. Liver

##### Liver Contrast-Enhanced Ultrasound and Heart Failure

Several Studies were performed [84,85,86,87,88,89,90] with different contrast agents in evaluating the diagnosis and staging of cirrhosis and portal hypertension (Table A3 in Appendix A): the first was performed with LEVOVIST^TM^ [84,86,88]; several studies with SONOVUE^TM^ [88,89]; and a more recent study with SONAZOID^TM^ [90]. The real-time characteristics of hepatic microvasculature, as a marker of the degree of chronic liver disease, have been studied since 1999 [84] with a simple, bedside examination: contrast-enhanced ultrasound (CEUS).

The foundational principle (Table A1, in Appendix A) established by Albrecht (1999) [84] demonstrated that a bolus of ultrasound contrast agent (initially Levovist^TM^) arrives significantly earlier in the hepatic veins of cirrhotic patients compared to those with non-cirrhotic liver disease or healthy controls.

A second study was published in 2002 [85]. This study investigates the early appearance of a contrast agent in the hepatic vein of cirrhotic patients compared to healthy individuals and those with hepatitis. The research aimed to determine if this early contrast arrival is due to intrahepatic or extrahepatic factors. After administering the contrast agent, the results showed that while arrival times in the hepatic artery and portal vein were similar across all groups, hepatic vein arrival times were significantly earlier in cirrhotic patients (median 18 s) compared to those with hepatitis (median 30 s) and healthy controls (median 31 s). The findings suggest that the earlier contrast arrival in cirrhosis is primarily due to intrahepatic hemodynamic changes, not extrahepatic factors.

The same group in 2003 published a study [86], which builds on a previous pilot investigation, that identified early microbubble arrival in the hepatic vein as a sensitive indicator of cirrhosis. The aim was to determine whether this index could also help grade the severity of diffuse liver disease. Microbubble injections were followed by a saline flush, and time–intensity curves of hepatic vein and carotid artery Doppler signals were analyzed. Hepatic vein transit time (HVTT) and carotid delay time (CDT) were calculated to assess liver function. The results showed that both HVTT and CDT decreased significantly as liver disease worsened: these differences were highly significant (*p* < 0.001). HVTT < 24 s and CDT < 10 s showed 100% sensitivity for detecting cirrhosis, but were not entirely specific, as some patients with fibrosis had values below these thresholds.

This “hepatic vein arrival time” (HVAT) became a cornerstone metric. Subsequent studies consistently validated this finding, with Giuseppetti (2004) [91] and Abbattista (2008 [92]; 2016 [93]) confirming that an HVAT cutoff of approximately 17 s could discriminate cirrhosis with high sensitivity (91–100%) and specificity (93–100%). The evolution from first-generation (Levovist^TM^) to second-generation (SonoVue) agents, as compared by Lim (2006) [88], further refined the technique, offering shorter transit times and excellent reproducibility.

A key advancement was the measurement of “intrahepatic transit times,” such as the interval between hepatic artery and hepatic vein enhancement (HA-HVTT). Several authors [87,94,95,96,97] showed that these intervals shorten progressively with the severity of fibrosis and cirrhosis, offering a quantitative means to stage liver disease. The same conclusions were also obtained by Li et al. [98] in hepatitis B patients. The utility of CEUS extends beyond mere diagnosis to the evaluation of its major complication, portal hypertension. Jeong (2015) [99] found that the intrahepatic transit time (ITT) was the most accurate CEUS parameter for predicting severe portal hypertension (HVPG ≥ 12 mmHg), with an AUC of 0.94. More recently, Zocco (2023) [100] combined CEUS with elastography, finding that liver parenchyma peak intensity (PI-LP) could predict clinically significant portal hypertension with perfect accuracy (AUC 1.000), while Zhou (2025) [101] demonstrated the value of spleen-specific CEUS parameters for predicting HVPG non-invasively.

Crucially, CEUS possesses inherent advantages that position it as an ideal tool for monitoring and accessible hemodynamic assessment. It is a dynamic examination, providing immediate physiological feedback. The procedure is quick to perform, costs relatively little compared to cross-sectional imaging, and can be performed portably at the patient’s bedside. It is important to note that, like all ultrasound techniques, it is operator-dependent and can be limited by patient body habitus, a factor less relevant for MRI [38]. It is a dynamic examination, providing immediate physiological feedback. The procedure is quick to perform, costs relatively little compared to cross-sectional imaging, and, most importantly, can be performed portably at the patient’s bedside. This makes it exceptionally accessible for frail, hospitalized, or frequently monitored patients. However, CEUS is not currently used routinely for the assessment of congestive hepatopathy (CH). While CEUS may help identify signs of portal hypertension by analyzing vessel interval times, regional perfusion, and vessel opacification patterns, it does not reveal specific diagnostic patterns for CH.

##### Liver Stiffness and Heart Failure

Liver elastography, a non-invasive method, is emerging as a valuable tool for assessing the increased venous congestion typically seen in heart failure. Research has indicated its potential as a prognostic marker, especially in patients with acute decompensated heart failure (ADHF) and those with left ventricular assist devices (LVADs). Millonig et al. [102] were pioneers in demonstrating a relationship between FibroScan liver stiffness measurements (LSMs) and central venous pressure in an animal model using Landrace pigs. They observed a consistent rise in LSM with increasing intravenous pressure (r = 1, *p* < 0.01). Colli et al. [103] conducted the first human study in 2010, showing that patients with acute decompensated heart failure typically exhibit elevated liver stiffness, which decreases as their condition improves, similar to NT-proBNP levels. Significant reductions in median liver stiffness (*p* < 0.003) and NT-proBNP levels (*p* < 0.001) were noted during hospitalization, while no significant changes were observed in control patients. In 2012, Hopper et al. [104] found that LSM did not significantly fluctuate with acute volume changes but was elevated in patients with various cardiac dysfunctions, including left-sided heart failure (LHF), right-sided heart failure (RHF), and ADHF (Table 1). Additionally, changes in volume status through diuresis did not significantly alter baseline LSM in ADHF patients. Similarly, Jalal et al. [105] identified a correlation between LSM and central venous pressure (CVP) in patients with congenital heart disease (CHD), reinforcing the idea that LSM could serve as a non-invasive marker of hepatic congestion in heart failure contexts. The relationship between liver elastography and passive venous congestion has thus been highlighted, positioning LSM as a potential marker for hepatic congestion in heart failure. Multiple studies have shown that elastography can serve as a non-invasive substitute for CVP measurement, as confirmed by right heart catheterization (RHC) (Table 1). Yoshitani et al. [106] found a positive correlation using ARFI, with CVP emerging as an independent predictor of increased LSM in multivariate analysis. Both Nishi et al. [107] and Potthoff et al. [108] studied heart failure patients post-LVAD placement, using FibroScan and ARFI, respectively, and found a linear relationship between pre-operative CVP and pre-operative LSM. Furthermore, Jalal et al. [105] demonstrated a correlation between CVP measured by RHC and LSM obtained via FibroScan in both adult and pediatric patients with congenital heart disease. This underscores the potential of LSM as a marker of hepatic congestion in the context of heart failure (Table 1). A recent meta-analysis by Macerola [109] confirmed that increased LS is associated with poor prognosis in patients hospitalized for HF and might help effectively identify those patients at high risk for worse outcomes.

**Liver stiffness measurements as a surrogate marker for central venous pressure**

Numerous studies have shown that elastography serves as an effective non-invasive alternative for assessing central venous pressure (CVP), typically measured through right heart catheterization (RHC) (Table 1). Yoshitani et al. [106] identified a positive correlation using acoustic radiation force impulse (ARFI), with CVP emerging as an independent predictor of increased liver stiffness measurements (LSMs) in multivariate analysis. Similarly, Nishi et al. [107] and Potthoff et al. [108] both examined heart failure patients following LVAD placement, using FibroScan and ARFI, respectively, and found a linear relationship between pre-operative CVP and pre-operative LSM.

Beyond the heart failure patient group, Jalal et al. [92] also discovered a correlation between CVP measured by RHC and LSM obtained via FibroScan in both adult and pediatric patients with congenital heart disease. These studies consistently demonstrated a baseline correlation between RHC and LSM, establishing LSM as a reliable non-invasive method for evaluating hydrostatic pressure. Few studies, however, have compared the correlations between CVP and other laboratory or echocardiographic markers of elevated pressure in conjunction with LSM. For instance, Taniguchi et al. [96] found that FibroScan offered greater sensitivity and accuracy in detecting right atrial pressure (RAP) above 10 mmHg compared to inferior vena cava (IVC) measurements obtained via echocardiography. Additionally, Jalal et al. [105] reported that LSM had a stronger correlation with CVP than with brain natriuretic peptide (BNP) (Table 1).

**I.**  **Clinical Application of liver stiffness evaluation in HF**

The existing data illuminate three key applications of liver stiffness evaluation:

(1) Assessment of adequate venous decongestion prior to discharge;
(2) Prognosis after an acute exacerbation;
(3) Risk stratification for determining right ventricular support needs before LVAD placement.

**II.**  **Elastography can demonstrate decongestion in acute decompensated heart failure**

Several studies have compared liver stiffness measurements (LSMs) at admission and discharge in patients with acute decompensated heart failure (ADHF). Most of these studies found a significant decrease in LSM after diuresis (Table 2). One study [104] showed that patients with ADHF had higher LSM compared to those with stable heart failure (HF) and normal cardiac function, with LSM correlating with markers like NT-proBNP, right atrial pressure, and right ventricular pressure. LSM was found to decrease significantly after diuresis, aligning with levels seen in stable HF patients. LSM also appeared to be a more accurate indicator of decongestion compared to other non-invasive markers.

Likewise, Alegre et al. [112] obtained significantly higher FibroScan LSM in ADHF patients as compared with stable biventricular HF patients [112]. LSM significantly decreased in the ADHF group after diuresis, reaching comparable levels to the stable HF groups [median 8.2 (5.1–11.2) kPa vs. median 6.5 (5.0–10.8) kPa] [112]. When compared with other non-invasive markers of CH, LSM more accurately demonstrated decongestion. Yoshitani et al. [106] related total bilirubin, AST, ALT, and GGT before and after diuresis and found no significant change, whereas body weight, LSM, and BNP all significantly decreased. While CVP was shown to be an independent predictor for changes in LSM, other markers of decongestion (body weight, BNP, and PCWP) did not correlate with changes in LSM despite significant improvement [106]. Two studies [103,104] established that LSM and NTproBNP were both significantly reduced after sufficient diuresis. In a cross-sectional study, Hopper et al. [104] determined that increased LSM correlated with increased bilirubin, GGT, and alkaline phosphatase in LHF, RHF, and ADHF groups. This was also the only study that showed a correlation of median LSM and NTproBNP (r = 0.24, *p* = 0.01), but failed to show a significant change in LSM after adequate diuresis. Together, these data confirm the known clinical course of CH, in which liver markers vary and are typically unreliable despite larger shifts in body volume, and support LSM as a superior tool. The inability to determine accurate reference ranges to assess for adequate decongestion is a major limitation. While most studies revealed a significant decrease in LSM after diuresis, a standard has not been established, and it is unclear if residual abnormalities in LSM reflect congestion or underlying fibrosis. As such, there may be discordance between liver stiffness and congestion in heart failure patients. Changes in LSM with therapy may reveal more beneficial than the absolute value of any particular measurement, as it is not possible to distinguish if high values represent congestion or fibrosis. Future studies should focus on individualized use of LSM to establish baseline diuresis goals for each patient prior to safe discharge.

**III.**  **Liver stiffness measurements as a prognostic tool in acute decompensated heart failure**

Elastography has been used by several research groups (Table 1) to predict the prognosis of patients with acute decompensated heart failure (ADHF), identifying liver stiffness measurement (LSM) as an independent predictor of adverse events. In a study by Saito et al. [100], ADHF patients were divided into low and high LSM groups, with higher LSM linked to increased rates of cardiovascular deaths and heart failure readmissions. High LSM was found to be the only independent risk factor for these events, surpassing echocardiographic and serologic data.

Similarly, Omote et al. [114] used ARFI to assess prognosis, finding that high LSM was independently associated with a greater risk of adverse events. Taniguchi et al. [115] found that higher discharge LSM levels were linked to worse outcomes, with LSM > 10.1 kPa being identified as an optimal cut-off for predicting short-term cardiac events. This study highlighted the value of LSM in improving prognostic models for heart failure.

Soloveva et al. [113] demonstrated that higher LSM at admission and discharge was associated with a higher likelihood of negative outcomes, including heart failure readmission and all-cause death. While LSM did not consistently correlate with liver pathology, it was effective in predicting adverse events, making it a useful tool for assessing decongestion before discharge.

Both Soloveva et al. [113] and Saito et al. [116] observed higher levels of total bilirubin and found no significant variation in BNP levels among patients with elevated LSM, while Taniguchi et al. [115] reported contrasting results. Soloveva et al. [113] and Taniguchi et al. [115] highlighted that LSM has a strong potential for predicting adverse events, indicating its importance as a clinical tool to evaluate decongestion before discharge. However, since elastography cannot distinguish between ongoing congestion and pre-existing fibrosis, further research is needed to understand the factors that make LSM a valuable prognostic tool.

Despite LSM’s inability to differentiate between residual congestion and underlying fibrosis, its role in risk stratification for urgent interventions, such as transplant referral, is emphasized. Future studies will focus on refining the timing of LSM measurements and establishing appropriate cut-off values for risk stratification (Table 3).

**IV.**  **Liver stiffness measurements as a prognostic tool after left ventricular assist device placement**

Potthoff et al. [108] first reported that liver stiffness measurement (LSM) using ARFI elastography showed a positive correlation with central venous pressure (CVP) and a significant post-operative decrease after left ventricular assist device (LVAD) placement, indicating improved CVP. Higher LSM was observed in patients who later died, suggesting its potential as a prognostic tool. Nishi et al. found that LSM was significantly higher in LVAD candidates who required right ventricular assist devices (RVADs), with a cut-off of ≥7.0 kPa indicating an increased need for RVADs. Patients with major adverse events (MAEs) had significantly higher LSM, with those having LSM ≥ 12.5 kPa experiencing more MAEs.

Nishi et al. [107] established that LSM was significantly higher in patients who also required RVADs. ROC analysis identified a cut-off ≥ 7.0 kPa for increased RVAD need.

Kashiyama et al. [111] identified that pre-operative LSM ≥ 12.8 kPa predicted right heart failure (RHF) after LVAD placement. Pre-operative LSM and left ventricular ejection fraction (LVEF) were independent risk factors for RVAD need, with a higher LSM cut-off of ≥14.0 kPa. This study highlighted LSM as an independent risk factor for adverse outcomes, enhancing existing prognostic tools for LVAD patients. This study also demonstrated the potential for non-invasive risk calculators, based on a subset of imaging and serologic tests, as an alternative to invasive procedures. However, this is the only published study assessing this relationship, and further research is needed.

Kobalava et al. [117] assessed the prognostic value of changes in congestion markers from admission to discharge in patients with irreversible heart failure. Liver stiffness, NT-proBNP, and lung ultrasound (LUS) were independent predictors of adverse outcomes. Wang et al. [118] found that LSM > 6.9 kPa and low “tricuspid annular plane systolic excursion” (TAPSE) could predict adverse cardiac events over two years in heart failure patients.

Misaka et al. [51] used shear wave elastography (SWE) to assess spleen tissue in 232 heart failure patients, finding that higher spleen elasticity and viscosity were associated with worse cardiac outcomes and lower event-free survival rates. Both spleen SWE and shear wave dispersion (SWD) were independent risk factors for adverse cardiac events, particularly in patients with impaired right ventricular function.

**V.**  **New Frontiers in LSM and HF**

Van Kleef et al. [119] examined liver stiffness in a cohort of 4153 individuals from the general population, both with and without chronic heart failure (CHF). They found that liver stiffness greater than or equal to 8.0 kPa was linked to an increased risk of mortality (aHR: 1.37, 95% CI: 1.00–1.89). However, this association was primarily observed in participants with heart failure (aHR: 2.48, 95% CI: 1.15–5.35), as elevated liver stiffness did not correlate with higher mortality in those without heart failure or coronary heart disease (CHD) (aHR: 1.07, 95% CI: 0.70–1.64). Thus, while high liver stiffness was associated with an increased mortality risk, this risk was driven by individuals with heart failure and remained significant even after excluding patients with viral hepatitis or alcohol abuse. In contrast, there was no significant or clinically relevant increase in mortality among individuals with high liver stiffness who did not have heart failure or CHD.

More recently, Yang et al. [120] conducted the first study to establish a correlation between heart failure with reduced ejection fraction (HFrEF) and liver stiffness. They used shear wave elastography (SWE) to assess liver stiffness, which is typically associated with the degree of fibrosis in liver diseases. The study found that patients with HFrEF had higher liver stiffness measurements (LSMs) of 8.4 ± 2.0 kPa and 10.3 ± 2.7 kPa, respectively. The LSM in HFrEF patients was significantly higher than in those with heart failure with mid-range ejection fraction (HFmrEF), and liver stiffness was even more elevated in patients with right heart failure (RHF) compared to those with HFmrEF and HFrEF. Additionally, other clinical parameters were abnormal only in patients with RHF and HFrEF. In summary, SWE appears to be a valuable clinical tool for the noninvasive evaluation of liver stiffness in patients with CHF, potentially aiding in the management of their treatment plans.

#### 3.1.2. Spleen

##### Splenic Contrast-Enhanced Ultrasound and Heart Failure

The spleen is now recognized as a central organ in heart failure (HF) pathophysiology, functioning as part of a bidirectional “cardio–splenic axis” where cardiac dysfunction induces splenic changes, and the spleen subsequently influences systemic inflammation and blood volume, impacting cardiac performance [121]. This axis is crucial post-myocardial infarction (MI), where the spleen actively coordinates inflammation and repair by releasing leukocytes and biosynthesizing specialized pro-resolving mediators (SPMs) like resolvins and lipoxins, which signal inflammation termination and promote tissue healing [122,123]. Disruption of this “splenocardiac axis”—via drugs (e.g., carprofen) or genetic deficiencies in resolution receptors (e.g., ALX/FPR2)—leads to non-resolving inflammation, adverse cardiac remodeling, and HF progression [124,125,126]. The effectiveness of this resolution system is modulated by lifestyle; omega-3 fats and exercise support SPM production, while an omega-6-rich diet promotes inflammation [127,128]. Critically, lipoxygenase (LOX) enzymes (5-LOX, 15-LOX) in the spleen act as pivotal regulators, catalyzing the production of either pro-inflammatory leukotrienes (from n-6 fatty acids) or pro-resolving SPMs (from n-3 fatty acids). The balance of this LOX-driven program determines cardiac repair outcomes after injury [129].

Clinical Implications and Role of CEUS: The spleen’s status provides a window into a patient’s HF state, reflecting both congestion from elevated venous pressure and systemic inflammation. Imaging techniques, particularly contrast-enhanced ultrasound (CEUS), are emerging as promising bedside tools for assessing splenic microvascular perfusion and congestion [130,131]. This builds on evidence that splenic Doppler and elastography indices correlate with haemodynamic parameters like right atrial pressure [132,133].

Several potential clinical applications for splenic CEUS in HF are identified:

Diagnostic Adjunct: To quantitatively assess organ congestion by revealing perfusion deficits indicative of splanchnic hypoperfusion or venous stasis, potentially correlating with invasive haemodynamics or congestion biomarkers.

Treatment Monitoring: To dynamically visualize improvement in splenic microcirculation in response to therapies like diuresis, providing an organ-level marker of effective decongestion.

Prognostic Biomarker: Integrating quantitative perfusion indices with risk models to improve patient stratification by capturing the combined impact of haemodynamic congestion and systemic inflammation [121].

Challenges and Future Directions: Pathophysiological causality (passive congestion vs. active neuroimmune remodeling) remains unresolved and requires longitudinal multimodal studies [121]. Significant barriers exist, including a current lack of high-quality, HF-specific evidence for splenic CEUS, as most existing literature focuses on focal lesions or uses other ultrasound modalities [41,130,132]. Technical standardization is critical for clinical adoption, requiring validated HF-specific protocols for acquisition (e.g., bolus vs. infusion, region-of-interest selection) and quantitative analysis, guided by general frameworks like the EFSUMB/WFUMB recommendations [56]. Confounding factors like body habitus and portal hypertension must also be accounted for.

In conclusion, splenic CEUS is a scientifically plausible and feasible bedside tool that uniquely interrogates microvascular perfusion, complementing other ultrasound measures. However, targeted clinical studies—standardizing protocols, establishing correlations with invasive haemodynamics, and validating prognostic utility—are essential to define its role. Given the spleen’s dual role in haemodynamics and immunity, CEUS may ultimately provide an integrated biomarker for contemporary HF care [121].

##### Splenic Stiffness and Heart Failure

The management of heart failure (HF) has progressively broadened from a narrow focus on the heart itself to a systemic appraisal of congestion and its far-reaching consequences. While the prognostic power of liver stiffness, measured by shear wave elastography (SWE), is firmly established in acute decompensated HF (ADHF) [109], clinical and research attention is now turning decisively towards a once-overlooked organ: the spleen. Emerging as a pivotal hub within the cardio–visceral axis, splenic SWE—quantifying spleen stiffness (SS)—is transcending its role as a simple marker of passive congestion. It is increasingly viewed as an integrated biosensor, reflecting a complex interplay of haemodynamic stress, pervasive immune activation, and the pathogenesis of debilitating patient-centric conditions like frailty. This multifaceted perspective promises a more holistic, mechanistically informed understanding of advanced HF, with potential to refine both risk stratification and therapeutic monitoring [51,134]. Technically, SS captures alterations in tissue tension arising from venous congestion, interstitial edema, and cellular remodeling [51,134]. Its measurement, however, demands rigorous standardization of probe position, depth, and breathing, as underscored by international elastography guidelines—a necessary step in adapting liver-centric protocols to the spleen in HF populations [103]. Pathophysiologically, an elevated SS is not a monolithic finding. It likely represents a confluence of passive and active processes. Passive venous congestion from elevated right-sided heart pressures engorges the splenic sinusoids with blood and interstitial fluid, increasing tissue turgor. Simultaneously, the spleen functions as a dynamic immune reservoir; chronic inflammation in HF can mobilize monocytes and remodel the splenic architecture through cellular infiltration and matrix changes, actively altering its mechanical properties independent of fluid pressure [79,121]. Therefore, a high SS in a patient with ADHF may signify haemodynamic congestion, heightened systemic inflammation, or, most probably, a pernicious combination of both. The clinical evidence underpinning SS is compelling and narrates the spleen’s evolving story in HF. Foundational work established its role in congestion: Saito et al. (2020) [134] demonstrated that SS directly correlates with invasively measured right atrial pressure (RAP) and, more importantly, that SS at hospital discharge is an independent predictor of death or rehospitalization, positioning it as a robust prognostic marker of residual splenic congestion [134]. Yet, the narrative extends beyond passive engorgement. Research by Hiraiwa et al. (2022) [135] on splenic volumetry revealed a biphasic response: a low splenic volume index (indicating a contracted spleen) was associated with forward failure, high systemic vascular resistance, and neurohormonal activation, also predicting poor outcomes [135]. This duality frames the spleen as a sensitive barometer of the circulatory system’s overall state. The most profound shift in understanding comes from research elucidating the active “cardio–splenic axis.” The landmark prospective study by Misaka et al. (2023) [51] showed that both SS and shear wave dispersion (SWD, a measure of tissue viscosity) were powerful independent predictors of hard cardiac outcomes. Crucially, they provided a mechanistic link, associating elevated SS with upregulation of CD36 mRNA in circulating leukocytes—a key molecule in monocyte activation [51,136]. This positions the spleen not as a passive victim of congestion but as an active participant in a maladaptive neuro-immune dialogue, serving as a reservoir for pro-inflammatory cells that fuel myocardial remodeling. Further illuminating this complex axis, Perrotta et al. described a sophisticated neural reflex where cardiac stress signals via the brainstem to the spleen, triggering the release of Placental Growth Factor (PlGF), which in turn stimulates protective macrophage proliferation in the heart, enabling adaptive remodeling. Disruption of this circuit leads to failure [137,138,139]. The relevance of splenic assessment further extends into the realm of patient functional status. Ishikawa et al. (2025) [140] connected reduced splenic venous flow to both frailty and low muscle mass in older cardiovascular patients, linking impaired splenic perfusion to the inflammatory and metabolic pathways that drive the wasting continuum from sarcopenia to cardiac cachexia [140,141]. This expands SS’s utility from a pure haemodynamic metric to a biomarker of systemic vulnerability. From a practical standpoint, measuring SS via point-SWE, 2D-SWE, or transient elastography is technically feasible [142]. However, its journey into routine clinical practice faces hurdles. The evidence base, while promising, is heterogeneous in methodology and patient populations, complicating the establishment of universal cut-off values. Technical confounders like splenic size and portal hypertension must be controlled [103]. Most critically, the causal interpretation of elevated SS remains ambiguous: does it primarily reflect reversible congestion or fixed inflammatory remodeling? Resolving this requires longitudinal studies pairing SS with invasive haemodynamics and inflammatory biomarkers [51,134]. The future trajectory of splenic SWE in HF hinges on concerted efforts toward standardization of acquisition protocols, validation of its incremental prognostic value over existing tools, and intervention studies tracking SS dynamically during therapy. Ultimately, the greatest insight will likely come from multimodal approaches that combine SS with perfusion imaging, volume analysis, and immune profiling [51,121,134]. If these challenges are met, spleen stiffness may well mature into an indispensable, non-invasive biomarker, offering a unique window into the intertwined haemodynamic and immune disturbances that define the advanced heart failure syndrome.

#### 3.1.3. Intestinal Wall

##### Intestinal Contrast-Enhanced Ultrasound and Heart Failure

Actually, intestinal contrast-enhanced ultrasound (CEUS) is not a primary diagnostic tool for heart failure, but it can be used to assess the health of the intestine in patients who also have heart failure, particularly to evaluate intestinal perfusion and inflammation. Heart failure can lead to reduced blood flow to the intestines [143,144], causing intestinal ischemia, which CEUS can help identify and differentiate from inflammatory conditions like inflammatory bowel disease (IBD) [145]. Based on the provided literature, heart failure (HF) plays a significant role in promoting vascular and functional alterations within the bowel, establishing a detrimental cycle via the “gut–heart axis.” In heart failure, impaired cardiac function leads to reduced systemic perfusion and venous congestion. This directly impacts the intestines by compromising intestinal microcirculation [12]. The resulting hypoperfusion and congestion cause oxidative stress and ischemia in the gut wall, which damages the delicate structure of the intestinal mucosa. This damage impairs the integrity of the intestinal barrier, making it “leaky” [146]. This breach in barrier function is a critical step. A leaky gut allows for bacterial translocation, where bacteria and their products, such as lipopolysaccharides (LPSs), pass from the gut lumen into the systemic circulation [147,148]. These microbial products then trigger a systemic inflammatory response, characterized by the activation of inflammatory pathways like the NLRP3 inflammasome. This inflammation, fueled by gut-derived toxins and metabolites like trimethylamine N-oxide (TMAO), further contributes to endothelial dysfunction and vascular damage, thereby exacerbating the progression of heart failure itself [14]. Thus, heart failure-induced vascular alterations in the bowel initiate a vicious cycle of increased gut permeability, systemic inflammation, and worsening cardiac function.

##### Intestinal Stiffness and Heart Failure

Heart failure (HF) is increasingly recognized as a systemic illness that significantly impacts the gastrointestinal tract. In congestive HF, elevated central venous pressure is transmitted to the splanchnic circulation, leading to intestinal wall edema, reduced perfusion, and impaired motility, which are associated with symptoms like early satiety and abdominal discomfort [149,150]. These structural changes suggest the bowel undergoes measurable alterations in HF. The rationale for using elastography to measure intestinal stiffness is drawn from analogous liver studies. Liver stiffness, quantified via shear wave or transient elastography, reliably increases with venous congestion in acute HF and decreases with effective decongestion, capturing reversible hemodynamic changes rather than fibrosis [151]. It is hypothesized that similar technology could detect congestion-related stiffness in the bowel wall. Furthermore, the gut–heart axis provides a mechanistic link. Intestinal congestion and edema can compromise the gut barrier, promoting bacterial translocation and systemic inflammation, as evidenced by elevated markers like fecal calprotectin in acute HF [152]. Since tissue stiffness can rise with edema and inflammation, intestinal elastography could theoretically serve as a non-invasive marker of gut dysfunction and systemic inflammatory burden. However, direct evidence is currently lacking. The intestine poses unique technical challenges for elastography due to peristalsis, layered anatomy, and luminal variability, with no standardized protocols yet established. Despite these hurdles, the pathophysiological convergence suggests intestinal stiffness measurement is a promising future research avenue for monitoring cardiosplanchnic interactions in HF.

#### 3.1.4. Kidney

##### Kidney Contrast-Enhanced Ultrasound and Heart Failure

The kidneys occupy a central role in the pathophysiology and clinical course of heart failure (HF). Hemodynamic derangements, neurohormonal activation, and inflammatory signaling interact to produce the cardiorenal syndrome, in which renal perfusion and venous congestion each contribute to declining glomerular filtration and adverse outcomes.

The kidneys play a central role in the pathophysiology of heart failure (HF), with hemodynamic, neurohormonal, and inflammatory interactions defining the Cardiorenal Syndrome (CRS)—a state of bidirectional organ dysfunction that worsens prognosis. The syndrome is systematically classified into five distinct subtypes [153].

Type 1 (Acute Cardiorenal Syndrome): An acute cardiac event, such as decompensated HF or cardiogenic shock, precipitates acute kidney injury (AKI) via reduced perfusion, venous congestion, and neurohormonal activation, significantly increasing morbidity and mortality [154,155].

Type 2 (Chronic Cardiorenal Syndrome): Chronic HF leads to progressive renal dysfunction from sustained low cardiac output, venous congestion, and neurohormonal activation, causing structural kidney damage and serving as a powerful predictor of adverse outcomes [156,157].

Type 3 (Acute Renocardiac Syndrome): An acute kidney injury induces acute cardiac dysfunction through volume overload, electrolyte disturbances, uremic toxins, and inflammation, rapidly impairing myocardial function [158,159].

Type 4 (Chronic Renocardiac Syndrome): Chronic kidney disease (CKD) promotes long-term cardiovascular injury, including left ventricular hypertrophy and arterial stiffening, driven by uremic toxins, mineral disorders, and chronic inflammation, leading to elevated cardiovascular mortality [160,161].

Type 5 (Secondary Cardiorenal Syndrome): A systemic condition like sepsis, diabetes, or cirrhosis simultaneously impairs both heart and kidney function through complex multisystem mechanisms [162,163]. The evolving understanding of heart failure as a multisystem disorder has sharpened the focus on organs beyond the heart, with the kidneys standing as critical sentinels of systemic hemodynamic and inflammatory stress. In this context, imaging the renal microcirculation offers an attractive, yet historically elusive, window into pathophysiological processes central to the cardiorenal syndrome. Contrast-enhanced ultrasound (CEUS) has emerged as a particularly promising modality for this task. By utilizing intravascular, gas-filled microbubbles, CEUS enables real-time, bedside assessment of parenchymal perfusion with high temporal resolution. Its safety profile—devoid of ionizing radiation or nephrotoxic contrast—makes it uniquely appealing for the repeated evaluations often required in patients with cardiac disease [56]. The technology and quantitative methods behind CEUS have matured significantly. International guidelines now outline protocols for its non-hepatic use, including both bolus and infusion techniques that generate time–intensity curves. From these analyses, parameters such as time-to-peak (TTP) and perfusion index (PI) are derived, offering complementary insights into microvascular blood velocity and volume. Because microbubbles faithfully track capillary flow, these metrics are thought to directly reflect the microcirculatory alterations that underpin cardiorenal interactions [56,164]. Clinically, early explorations in HF populations, though limited, are illuminating. Studies have demonstrated that patients with chronic congestive HF exhibit altered renal cortical perfusion on CEUS—specifically, a prolonged TTP and reduced contrast intensity—patterns that partially normalize following decongestive therapy [165]. This provides tangible evidence that CEUS can detect reversible haemodynamic impairment. Earlier pilot work in critical care and perioperative settings has further cemented its feasibility for serial monitoring, showing dynamic perfusion changes in response to physiological and pharmacological interventions [166,167]. Broader observational studies link these CEUS-derived biomarkers to glomerular filtration rate and histologic severity, suggesting they capture clinically meaningful variation in renal microvascular health [168,169]. Mechanistically, CEUS probes two intertwined processes in HF: renal venous congestion, which reduces effective perfusion pressure and prolongs transit times, and neurohormonal vasoconstriction with microvascular rarefaction, which diminishes blood volume and velocity. The power of CEUS lies in its potential to serially distinguish the contributions of these processes at the bedside, offering a dynamic view of the kidney’s response to therapy [165,166,167]. The potential clinical utilities are compelling, spanning diagnostic, monitoring, and prognostic domains. CEUS could identify occult renal congestion, guide the titration of diuretic or vasodilator therapy by revealing organ-level perfusion responses, and potentially stratify patients at higher risk of adverse outcomes [168,169]. However, the path to routine clinical integration is not without obstacles. Significant technical heterogeneity exists in acquisition and analysis methods, physiological confounders abound, and inter-operator variability presents a challenge for standardization [56,169]. Perhaps most importantly, while CEUS sensitively detects perfusion changes, it cannot alone decipher whether these represent reversible congestion or irreversible parenchymal injury; this requires correlative data from biomarkers or invasive haemodynamics. Therefore, the future trajectory of renal CEUS in HF hinges on a concerted research agenda. This must prioritize the standardization of protocols, rigorous mechanistic correlation studies with invasive measurements, and prospective trials to validate its prognostic and therapeutic guidance value [56]. In summary, renal CEUS represents a scientifically plausible and technically feasible innovation that could bridge the gap between hemodynamic assessment and personalized kidney protection in cardiorenal medicine. Its promise is clear, but its ultimate role will be defined by our success in navigating the necessary steps of validation and standardization.

##### Kidney Stiffness and Heart Failure

The intricate interplay between the heart and kidneys, clinically enshrined in the concept of cardiorenal syndrome (CRS), forms a central pillar in the pathophysiology of heart failure (HF). Within this complex bidirectional relationship, where dysfunction in one organ accelerates decline in the other [170], the paradigm of renal impairment has evolved. The traditional focus on hypoperfusion has been broadened to recognize venous congestion as a primary driver of kidney dysfunction in decompensated HF. This concept of “congestive nephropathy” reframes renal injury as a consequence of elevated renal venous and interstitial pressures, potentially offering a reversible therapeutic target [171]. It is within this conceptual shift that the measurement of renal tissue stiffness via elastography emerges as a compelling investigative tool. By capturing a composite signal of congestion, edema, inflammation, and eventual fibrosis, renal elastography presents a non-invasive window into organ-level mechanical properties that could refine diagnosis, monitoring, and prognosis in HF. The technical foundation for this application is robust, having been established in nephrology through extensive research in chronic kidney disease (CKD). Meta-analytic evidence solidly validates the whear wave elastography (SWE) as a powerful tool for assessing CKD severity. The core principle is clear: SWE-measured renal stiffness shows a significant, reliable correlation with the degree of histologic fibrosis, the hallmark of progressive CKD. A key meta-analysis confirms high diagnostic accuracy, with a summary sensitivity of 84%, specificity of 80%, and an area under the SROC curve of 0.89 for detecting significant fibrosis [50]. This positions SWE as a promising, repeatable alternative to biopsy for monitoring disease progression. However, this same consolidating research underscores a critical barrier to widespread clinical adoption: profound heterogeneity. Variability springs from three main sources. First, differences between ultrasound devices and manufacturers lead to inconsistencies in absolute stiffness values [172]. Second, a lack of uniform acquisition protocols is problematic, exemplified by the use of different quantitative parameters (kPa vs. m/s) and a wide range of reported diagnostic thresholds—from as low as 4.31 kPa to as high as 22.95 kPa for detecting fibrosis [50,173,174]. Finally, the diversity of clinical populations studied, encompassing various CKD etiologies and stages, introduces inherent biological variability that single thresholds cannot adequately capture [172]. Interpreting renal stiffness, especially in the dynamic context of HF, requires navigating a complex pathophysiology. The signal is multifactorial. Acutely, elevated renal venous pressure increases interstitial fluid, raising tissue turgor and apparent stiffness without permanent injury—a purely haemodynamic effect [175]. Experimental work confirms that renal stiffness is exquisitely sensitive to perfusion changes, with both arterial inflow and venous outflow acting as dominant confounders [176]. Chronically, however, repetitive congestion and inflammation can incite genuine tubulointerstitial fibrosis, leading to permanent, irreversible stiffness elevations [176,177]. Thus, the central interpretive challenge in HF is distinguishing reversible, congestion-driven stiffness from fixed, fibrotic stiffness. Promisingly, clinical studies suggest this may be feasible; research in critically ill patients shows renal stiffness rises with fluid loading and can respond to hemodynamic interventions, supporting its potential as a bedside index of renal congestion [178]. This mechanistic understanding frames several plausible clinical roles for renal elastography in HF management, though these remain largely investigational. Diagnostically, an elevated stiffness in an HF patient with worsening renal function could help identify a “congestive nephropathy” phenotype, steering therapy toward aggressive decongestion rather than misattributing the injury to intrinsic renal disease [179]. For monitoring, the technique’s non-invasive, repeatable nature makes it ideal for tracking organ-level responses to diuresis or device therapy, potentially providing an early signal of renal recovery or ongoing stress. Prognostically, a stiffness measurement that integrates both acute haemodynamic stress and chronic structural vulnerability might improve risk stratification for persistent kidney injury or adverse clinical outcomes. Despite this compelling rationale, substantial caveats remain. Technical heterogeneity demands urgent standardization of acquisition protocols, region-of-interest selection, and reporting units [50]. The kidney’s anisotropic structure means measurements are sensitive to sampling location (cortex vs. medulla) and confounded by perfusion and intra-abdominal pressure [176]. Crucially, most validation data come from CKD populations, not HF cohorts, necessitating targeted studies that correlate stiffness with invasive haemodynamics (e.g., renal venous pressure) and biomarkers in HF patients [50,176,180]. Therefore, the path forward requires a concerted research agenda. Priority must be afforded to standardization initiatives to ensure reproducibility across centers. Mechanistic studies must integrate SWE with gold-standard haemodynamic and inflammatory phenotyping to deconvolute the stiffness signal. Ultimately, prospective cohort studies in HF are needed to validate whether renal stiffness independently predicts hard clinical endpoints, and pragmatic trials must assess whether stiffness-guided decongestion improves outcomes. In conclusion, renal stiffness measured by elastography is a biologically plausible biomarker sitting at the intersection of haemodynamics and tissue remodeling in HF. While validated as a correlate of fibrosis in CKD, its application in the oscillating environment of cardiorenal syndrome is more nuanced, charged with the potential to differentiate reversible congestion from irreversible injury. Its journey from a promising research tool to a reliable clinical asset hinges entirely on overcoming standardization hurdles and securing robust, HF-specific evidence that confirms its incremental value in guiding personalized cardiorenal care.

### 3.2. Congestion, Inflammation, and Fibrosis in the Heart

#### 3.2.1. Cardiac Contrast-Enhanced Ultrasound and Heart Failure

Heart failure (HF) remains a leading cause of morbidity and mortality worldwide, characterized by impaired ventricular function and structural remodeling that progresses despite modern therapies. Accurate imaging is central to diagnosis, risk stratification, and therapeutic decision-making in HF. Among imaging modalities, echocardiography remains the most widely employed, given its accessibility, non-invasiveness, and cost-effectiveness. However, a persistent limitation of standard echocardiography is suboptimal endocardial border delineation, observed in up to 30% of patients, particularly in those with obesity, lung disease, or distorted chest anatomy [181]. This diagnostic challenge can significantly impair assessment of left ventricular (LV) volumes, ejection fraction (LVEF), and wall motion, parameters that are crucial in guiding therapy. The introduction of contrast-enhanced ultrasound (CEUS), particularly with second-generation agents such as SonoVue^®^ (Bracco, Milan, Italy) and Optison^®^ (GE Healthcare, Boston, MA, USA), has revolutionized cardiac ultrasound by improving LV opacification, enhancing endocardial definition, and enabling evaluation of myocardial perfusion. These developments carry important implications for the management of HF patients.

##### Improvement in Endocardial Border Delineation and LV Function Assessment

The principal advantage of CEUS in HF lies in its ability to transform nondiagnostic studies into diagnostic ones. Inadequate visualization of the endocardial border not only reduces reproducibility but also introduces significant errors in LVEF estimation, which may affect eligibility for therapies such as implantable cardioverter defibrillators (ICDs) or cardiac resynchronization therapy. Multiple multicenter trials have demonstrated that intravenous contrast markedly improves endocardial visualization scores compared with tissue harmonic imaging alone, with the greatest benefit in apical segments. In one multicenter trial, SonoVue enabled a significant increase in correctly classified LVEF categories (normal, mildly reduced, or severely reduced) when compared with cardiac magnetic resonance (CMR), the reference standard [182]. Similarly, Yu et al. confirmed that CEUS yielded more accurate LV volumes and EF even in critically ill patients in intensive care units, where image quality is often poor [183]. This is particularly relevant in acute HF settings, where therapeutic decisions depend on rapid and reliable functional assessment. The clinical impact of improved reproducibility is non-trivial. Serial measurements of LV function are often required to monitor remodeling, response to therapy, or progression of HF. By reducing interobserver variability, CEUS strengthens confidence in echocardiography as a follow-up tool compared with modalities such as radionuclide ventriculography or CMR.

##### CEUS in Stress Echocardiography and Ischemic Heart Failure

A large proportion of HF cases are secondary to ischemic cardiomyopathy. Accurate detection of inducible ischemia and viability is vital in determining revascularization strategies. Stress echocardiography, whether pharmacologic (dobutamine, dipyridamole) or exercise-based, is widely used for ischemia detection. However, its diagnostic accuracy strongly depends on image quality, as interpretation relies on visualizing regional wall thickening and motion. Suboptimal visualization of even two LV segments can render stress echocardiograms nondiagnostic [184].

Contrast use during stress echocardiography has consistently reduced the proportion of inconclusive studies. Jung et al. demonstrated that with SonoVue, more than 95% of previously nondiagnostic stress echocardiograms became interpretable [185]. Beyond improving wall motion analysis, CEUS enables simultaneous assessment of myocardial perfusion by exploiting the unique acoustic properties of microbubbles. Experimental and clinical studies indicate that perfusion defects often precede wall motion abnormalities in the ischemic cascade, thereby enhancing sensitivity for early ischemia detection [182].

In HF patients with coronary artery disease (CAD), this dual assessment of function and perfusion is particularly valuable. Dobutamine stress CEUS has shown comparable sensitivity and specificity for CAD detection to single-photon emission computed tomography (SPECT), and in some cases, superior ability to delineate multi-vessel disease, which carries important prognostic implications [185]. Moreover, vasodilator CEUS has proven to be at least as accurate as SPECT in patients unable to undergo stress testing [186]. Thus, in ischemic HF, CEUS extends beyond a tool for LVEF assessment to become a powerful modality for risk stratification and therapeutic decision-making.

##### Applications in Structural Assessment and Thrombus Detection

HF patients frequently present with structural complications such as LV thrombi, aneurysms, or apical hypertrophic cardiomyopathy. Native echocardiography often struggles with near-field artifacts and suboptimal apical imaging, leading to underdiagnosis. CEUS provides unequivocal delineation of apical thrombi, outperforming both fundamental and tissue harmonic imaging [181]. This is clinically critical given the embolic risk associated with LV thrombus. Likewise, CEUS has clarified diagnoses of apical hypertrophic cardiomyopathy and LV non-compaction, entities with important prognostic and therapeutic consequences [181].

##### Safety, Contraindications, and Risk–Benefit Considerations

The safety of ultrasound contrast agents has been carefully scrutinized. Adverse reactions are generally mild, such as headache or flushing, though rare allergic and potentially life-threatening reactions have been reported. Large-scale post-marketing surveillance involving over 200,000 SonoVue vials revealed serious adverse events (SAEs) in only 0.01% of cases [181]. Nonetheless, temporal associations between SonoVue administration and deaths in patients with unstable CAD prompted the European Medicines Agency to issue contraindications for its use in acute coronary syndrome, unstable ischemic disease, acute decompensated HF (NYHA class III–IV), and severe arrhythmias [187]. It is noteworthy that these restrictions mirror the patient groups who would already be excluded from stress echocardiography due to inherent procedural risks. Importantly, large registries of dobutamine stress CEUS have not shown an increased risk compared with native stress echocardiography [187]. Compared with alternative modalities such as SPECT or invasive angiography, CEUS carries a lower risk of radiation exposure and contrast nephrotoxicity [187]. Therefore, when applied within appropriate clinical indications, CEUS demonstrates an excellent risk–benefit ratio, particularly when the consequences of missed diagnoses in HF are considered.

##### Emerging Technologies and Future Perspectives

Advances in low-power imaging modalities, such as pulse inversion and power modulation, have improved signal-to-noise ratios, reduced microbubble destruction, and enabled real-time assessment of myocardial perfusion. These refinements are especially promising for HF, where a comprehensive assessment of both structure and perfusion is desirable. Automated quantification algorithms are also under development, aiming to integrate CEUS into workflows that provide objective and reproducible measurements of regional wall motion and global LV function [181]. Additionally, contrast agents are being explored as targeted molecular probes, opening possibilities for imaging myocardial angiogenesis, inflammation, or fibrosis, all of which are central processes in HF pathophysiology. While these applications remain investigational, they highlight the evolving role of CEUS beyond conventional LV opacification.

#### 3.2.2. Cardiac Stiffness and Heart Failure

The clinical challenge of heart failure with preserved ejection fraction (HFpEF) has long been defined by a diagnostic paradox: a heart that contracts adequately but fails to relax, often due to increased myocardial stiffness driven by microscopic fibrosis [188]. For decades, assessing this fundamental property relied on indirect, inferential clues. The advent of shear wave elastography (SWE) now illuminates this once-elusive aspect of cardiac mechanics, offering a paradigm shift by providing a direct, quantitative measure of tissue stiffness, effectively translating the abstract notion of a “stiff heart” into a concrete, numerical biomarker. The principle is elegantly rooted in physics: focused ultrasound pulses generate minute shear waves within the myocardium. By tracking their propagation speed with ultrafast imaging, stiffness can be calculated in kilopascals (kPa)—stiffer tissue propagates waves faster. This provides a “virtual biopsy” of the heart’s mechanical environment, a significant advance over traditional methods that only infer stiffness from its hemodynamic consequences. Clinically, this translation has been profound. Seminal work by Villemain et al. (2019) [189] established that myocardial stiffness increases with healthy aging but is dramatically elevated in HFpEF and hypertrophic cardiomyopathy, with an 8 kPa cut-off effectively distinguishing disease. This offers an objective diagnostic tool for a notoriously ambiguous condition. The technology also shows promise in identifying early pathological remodeling. Research by Cvijic et al. (2020) [190] demonstrated elevated shear wave velocities in hypertensive patients, particularly those with concentric hypertrophy, suggesting an ability to detect the transition to pathological stiffening before overt HF develops. Perhaps most compellingly, SWE probes the very substrate of stiffness: fibrosis. Recent work by Petrescu et al. (2025) [191] directly correlated shear wave velocities with cardiac MRI measures of fibrosis, confirming that the technology can not only detect fibrosis but also differentiate between its interstitial and replacement forms. This non-invasive stratification could revolutionize prognostication and therapy monitoring. The application is expanding demographically, with studies like that of Youssef et al. (2024) [192] establishing pediatric norms and utility. Looking ahead, the technology is pushing into dynamic functional assessment. A pioneering pilot study by Venet et al. (2025) [193] explored calculating myocardial work by combining cyclic stiffness measurements with strain data, revealing abnormal work loops in pediatric cardiomyopathy and hinting at future assessments of regional cardiac efficiency. In conclusion, shear wave elastography represents a transformative advance in cardiac diagnostics by directly quantifying myocardial stiffness, the core defect in HFpEF. While questions of standardization and adoption remain [194], its capacity to provide an objective, tissue-level biomarker paves the way for more precise diagnosis, risk stratification, and personalized treatment strategies in heart failure.

### 3.3. Congestion, Inflammation, and Fibrosis in the Lung

#### 3.3.1. LUNG Contrast-Enhanced Ultrasound and Heart Failure

The clinical imperative in heart failure management lies in accurately assessing pulmonary congestion, a key driver of symptoms and adverse outcomes, while navigating the limitations of traditional tools like insensitive chest radiography or confounded natriuretic peptides [195,196]. In this diagnostic landscape, lung ultrasound (LUS) has firmly established itself as an indispensable point-of-care modality. Its principle exploits the acoustic physics of the lung: in health, ultrasound reflects off air-filled alveoli, creating A-lines, but interstitial edema fluid creates reverberation artifacts seen as vertical B-lines [197,198,199]. The burden of these B-lines correlates with extravascular lung water and filling pressures, providing a reliable, semi-quantitative measure of congestion with proven prognostic value, such as predicting poor outcomes post-discharge [200,201]. However, LUS primarily images the consequence of hemodynamic disturbance—accumulated fluid. A more sophisticated technique, contrast-enhanced ultrasound (CEUS), seeks to probe the underlying process by assessing the pulmonary microvasculature itself. Using intravascular microbubble tracers that do not extravasate [39], CEUS generates time–intensity curves (TICs) reflecting perfusion parameters like arrival time and wash-out kinetics [202,203]. Mechanistically, it is plausible that pulmonary congestion—with its capillary engorgement and interstitial compression—could alter these CEUS-derived metrics, producing delayed or blunted curves. Despite this intriguing hypothesis, the current clinical evidence firmly relegates CEUS to a secondary, complementary role in pulmonary congestion assessment. Its fundamental physical limitations are significant: microbubbles cannot image interstitial fluid directly, and the technique is acoustically obstructed by aerated lung, confining its view to pleural-based or consolidated areas [202]. Consequently, the robust literature validating CEUS in lung imaging almost exclusively addresses focal lesion characterization, not diffuse parenchymal disease, and studies have shown that individual TIC parameters like arrival time lack reliability for differentiating such lesions [202,204]. Therefore, CEUS cannot be recommended to supplant LUS for quantifying or monitoring diffuse pulmonary congestion. LUS remains the validated, pragmatic bedside standard for this purpose. Nonetheless, CEUS retains a definitive clinical role in evaluating concomitant focal lung pathology, such as consolidations or infarcts, in heart failure patients [44,202]. Furthermore, the prognostic utility of congestion assessment underscores the importance of LUS in identifying subclinical “hemodynamic congestion” post-discharge, a strong predictor of readmission [205,206], though standardization challenges remain [207]. In summary, the narrative of lung imaging in heart failure highlights a clear division of labor. LUS with B-lines is the workhorse for detecting and grading diffuse pulmonary congestion. CEUS, while a powerful research tool offering a unique window into microvascular perfusion, currently remains a speculative technique for diffuse congestion assessment, with its promising mechanistic hypotheses awaiting rigorous clinical validation. Their roles are complementary: LUS for the interstitial fluid, and CEUS for the vascular architecture when focal lung pathology raises diagnostic questions.

#### 3.3.2. LUNG Stiffness and Heart Failure

The assessment of pulmonary congestion in heart failure has evolved from inferring elevated pressures to directly visualizing extravascular lung water via lung ultrasound (LUS) B-lines [208,209]. Yet beyond fluid accumulation, the intrinsic mechanical stiffness of lung tissue itself represents a richer, integrative biomarker. This stiffness captures the combined effects of interstitial edema, alveolar collapse, surfactant dysfunction, and fibrotic remodeling, offering a potential link between hemodynamic status, gas exchange, and long-term disease trajectory in HF. Measuring lung stiffness non-invasively is technically demanding due to ultrasound scattering by air. However, several promising approaches have emerged. Ultrasound-based elastography techniques, such as surface wave and shear wave methods, estimate superficial lung stiffness by measuring the speed of mechanically induced waves across the pleura. These have been validated against CT and physiologic indices in interstitial lung disease [210,211,212]. Complementarily, magnetic resonance elastography (MRE) provides a volumetric measure of parenchymal shear modulus, with recent free-breathing sequences proving feasible in vivo, serving as a valuable research standard [213]. Pathophysiologically, lung stiffness in HF is dynamic and multifactorial. Acutely, elevated left-sided filling pressures cause interstitial and alveolar edema, increasing tissue turgor and stiffness, which directly reduces compliance and impairs gas exchange [214]. Chronic, persistent congestion and inflammation can drive fibroblast activation and extracellular matrix deposition, leading to irreversible fibrotic stiffening. Concurrent pulmonary vascular stiffening also occurs, worsening right ventricular afterload and mechanically interacting with parenchymal mechanics [215,216]. Early studies show these techniques have discriminative power. Lung ultrasound surface wave elastography (LUSWE) correlates with structural changes in interstitial lung disease and post-pneumonia fibrosis [210,212], while MRE confirms measurable, respiration-dependent stiffness in vivo [213]. Furthermore, pulmonary vascular stiffening is a recognized prognostic marker in left heart disease [215,216]. Significant caveats, however, impede immediate clinical adoption. Measurements are limited to superficial layers, missing deep pathology, and results are influenced by probe position, respiratory phase, and pleural coupling. Technical heterogeneity across elastography modes and vendors prevents universal cut-offs [210,211,212,217]. Common HF confounders like pleural effusions, chest wall edema, and obesity also affect readings. Crucially, elastography alone cannot distinguish reversible edema from fixed fibrosis, necessitating multimodal correlation with imaging, pulmonary function tests, and biomarkers. In conclusion, while lung stiffness measurement via elastography is a promising frontier for understanding cardiopulmonary interactions in HF—particularly in HFpEF—its transition from plausible research to clinical practice hinges on overcoming technical standardization hurdles and validating its incremental value in targeted HF cohorts.

### 3.4. The Utility of Splanchnic, Cardiac, and Thoracic CEUS and SWE in Heart Failure

Heart failure (HF) is increasingly recognized not as an isolated cardiac disorder, but as a systemic syndrome characterized by venous congestion, inflammatory activation, and progressive tissue remodeling that extends across multiple organ systems. The splanchnic organs—the liver, spleen, intestines, and kidneys—function as a high-compliance vascular reservoir, making them particularly vulnerable to the retrograde transmission of elevated central venous pressure. This venous hypertension triggers a cascade of congestion, endothelial dysfunction, and immune activation [1,2,3,4,6,7,12]. To capture this multisite pathophysiology, an integrated ultrasound framework has emerged, synthesizing two advanced, complementary techniques: contrast-enhanced ultrasound (CEUS) for quantifying hemodynamic impairment and shear wave elastography (SWE) for quantifying the resultant mechanical tissue changes. Together, they form a unified diagnostic strategy for characterizing the full spectrum of venous congestion.

#### 3.4.1. CEUS: The Functional Hemodynamic Pillar

CEUS operates by tracking intravascular microbubbles in real-time, providing a dynamic window into organ perfusion and microvascular transit. Its value lies in detecting the functional consequences of elevated venous pressure. In the liver, CEUS parameters like hepatic vein arrival time (HVAT) reliably shorten in proportion to congestion and portal hypertension, reflecting increased sinusoidal pressure and intrahepatic shunting. In the spleen, CEUS offers insight into the cardio–splenic axis, with altered perfusion kinetics correlating with portal pressure and systemic inflammation [84,86,87,90]. In the kidneys, CEUS identifies delayed cortical perfusion (increased time-to-peak), a direct marker of renal venous congestion contributing to cardiorenal syndrome [164,165,166,218]. In the thorax, while standard lung ultrasound B-lines indicate interstitial edema, CEUS assessment of pulmonary microvascular perfusion remains experimental but holds potential for evaluating capillary stress failure.

#### 3.4.2. SWE: The Structural Mechanical Counterpart

SWE complements CEUS by quantifying tissue stiffness, an objective metric of parenchymal health. In HF, increased stiffness can signal either acute, reversible congestion or chronic, irreversible fibrosis. Liver stiffness measurement (LSM) is the most established application. It strongly correlates with invasively measured central venous pressure [106,107,108,111,115], decreases with effective diuresis [104,108,112], and serves as a powerful independent predictor of adverse events, readmission, and mortality [110,113,114,116,219].

Splenic and renal SWE similarly identify congestion-related stiffness, with recent studies showing splenic SWE is independently associated with HF outcomes [51].

Thoracic SWE, though technically challenging due to air interference, is an emerging frontier for assessing lung parenchymal stiffness related to edema or fibrotic remodeling.

#### 3.4.3. The Iterative, Integrated Clinical Approach

**Figure 5:**
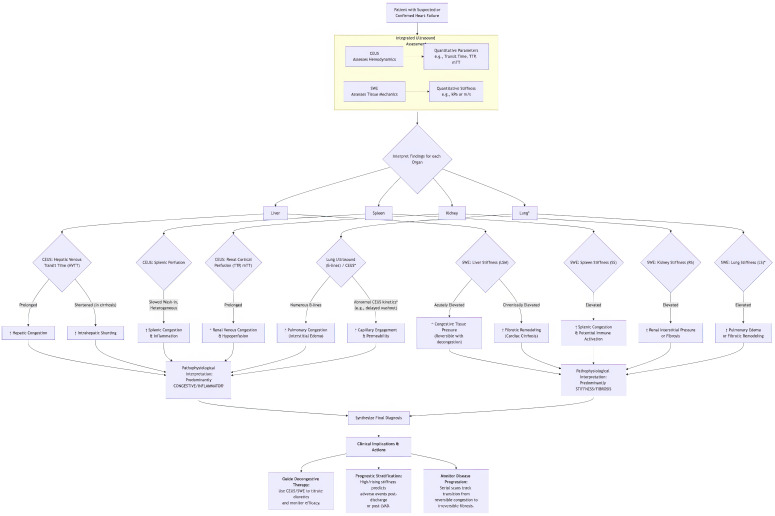
This figure illustrates an integrated ultrasound approach for assessing heart failure’s systemic effects. It combines the functional, hemodynamic insights from contrast-enhanced ultrasound (CEUS)—which reveals congestion via prolonged transit times—with the structural metrics from shear wave elastography (SWE)—which quantifies fibrotic tissue stiffness. Together, they guide therapy, stratify risk, and monitor for irreversible organ damage, while applications in lung assessment remain an emerging frontier. * (Only theoretical considerations).

The true diagnostic power is unlocked through the iterative and integrated application of these modalities alongside conventional ultrasound, creating a layered assessment cycle (Figure 5, Table 4).

Conventional ultrasound (B-mode/Doppler) serves as the foundational first tier. It rapidly assesses macrocirculatory hemodynamics at the bedside—such as IVC dilation or altered venous Doppler patterns—providing an immediate, gross evaluation of volume status [1].

CEUS forms the second tier, refining the picture by quantifying microvascular perfusion. It can detect subtle congestion and microvascular heterogeneity before Doppler changes are apparent, revealing the functional impact of venous hypertension at the capillary level. SWE completes the third diagnostic tier by adding crucial structural insight. It distinguishes reversible edema (which improves with therapy) from fixed fibrotic remodeling (which carries a worse prognosis), thereby connecting acute hemodynamic stress to long-term tissue consequences. Used serially, this framework creates a dynamic feedback loop for management. Clinicians can start with Doppler to detect congestion, use CEUS to quantify its microvascular extent, and employ SWE to gauge structural involvement. Treatment response can be monitored through normalization of Doppler patterns, shortening of CEUS transit times, and reduction in SWE stiffness. Critically, a persistently elevated SWE value despite improved hemodynamics signals irreversible damage, prompting a change in management strategy or prognostic outlook.

In conclusion, the integration of CEUS and SWE within a broader ultrasound protocol supports a multiparametric, organ-based approach to HF. This paradigm synthesizes complex pathophysiology into actionable diagnostics, enabling clinicians to distinguish reversible congestion from fixed fibrosis, guide decongestive strategies at the bedside, perform sophisticated risk stratification, and monitor for the transition to irreversible organ damage. While applications in splanchnic organs are advancing, pulmonary assessment remains an evolving frontier. Ultimately, this integrated framework embodies a modern, physiologically coherent approach to managing the systemic burden of heart failure.

## 4. Discussion

This work is the first review on the use of advanced ultrasound techniques (CEUS and SWE) in the study of splanchnic, cardiac, and thoracic vascularization in heart failure. HF is increasingly recognized as a systemic disease, involving complex interactions between hemodynamic overload, microvascular dysfunction, inflammatory activation, and organ remodeling. For too long, the clinical gaze in heart failure has been myopically fixed on the heart itself—its ejection fraction, its wall motion, its chamber sizes. Yet, anyone who manages this complex syndrome knows the story is far larger. Heart failure is a systemic disease, a narrative written across multiple organs through the intertwined plotlines of hemodynamic overload, microvascular dysfunction, and inflammatory signaling. The spleen, liver, kidneys, and lungs are not mere supporting actors; they are central characters whose subplots of congestion and remodeling critically determine the patient’s clinical course and outcome [220]. The search for a simple, non-invasive way to read this systemic story has been long-standing. We began with blood tests and scores. The FIB-4 index, for instance, hinted at a connection, showing an association with early heart failure markers independent of arterial stiffness [221]. Such serum-based tools proved useful and cost-effective for initial, broad screening, particularly in metabolic liver disease [222,223]. But they were limited, offering only indirect clues. A pivotal meta-analysis later confirmed what many suspected: for truly assessing tissue health, imaging-based techniques reign supreme. It showed that shear wave elastography (SWE) and MR elastography provided the highest accuracy for staging liver fibrosis [224]. CEUS has shown promise in detecting and characterizing portal vein thrombosis, particularly in hepatocellular carcinoma (HCC) [225]. Studies demonstrate CEUS’s superiority over CT in thrombus detection and characterization. Several Studies were performed [84,85,86,87,88,89,90] with different contrast agents in evaluating the diagnosis and staging of cirrhosis and portal hypertension. Actually, however, CEUS is not routinely used for CH assessment. While CEUS may help identify signs of portal hypertension by analyzing vessel interval times, regional perfusion, and vessel opacification patterns, it does not reveal specific diagnostic patterns for CH.

Conversely, SWE was a eureka moment for heart failure specialists: if SWE could so precisely map the fibrotic scars of liver disease, surely it could quantify the congestive swelling and scarring of a liver burdened by a failing heart. This realization shines a light on the limitations of our traditional bedside tool: conventional ultrasound. While indispensable—our review details its role in detecting a dilated IVC or abnormal venous Doppler patterns as signs of congestion [1]—it often feels like reading only the chapter titles. It shows us the gross, macrovascular consequences but misses the early microvascular drama and cannot distinguish between a tense, water-logged organ (reversible edema) and one hardened by scar tissue (irreversible fibrosis). This distinction is the cliffhanger at every discharge decision. Enter the two advanced techniques that form the core of this discourse: contrast-enhanced ultrasound (CEUS) and shear wave elastography (SWE). Think of them as complementary lenses, bringing different layers of the story into focus. CEUS is the functional lens. By tracking the passage of harmless microbubbles through an organ’s microvasculature, it lets us watch the flow of the narrative in real-time. A rapid transit of bubbles from artery to hepatic vein signals high pressure and shunting, a direct visual of venous congestion. In the spleen, peculiar perfusion patterns may reveal its activation in the inflammatory subplot of the cardio–splenic axis [84,86,87,90]. In the kidney, a delayed arrival of contrast to the cortex paints a picture of venous stasis, the very essence of congestive nephropathy [164,165,166,218]. CEUS detects the haemodynamic disturbance at its source, often before the plot escalates to overt clinical signs. SWE, in contrast, is the structural lens. It does not measure flow but rather the firmness of the stage itself. By sending gentle shear waves through tissue and measuring their speed, it quantifies stiffness in kilopascals. In the context of HF, this stiffness integrates the entire history of insult: the acute swelling from today’s fluid overload and the permanent scarring from years of chronic congestion. Its prognostic power is profound. Liver stiffness measured by SWE has been validated as a non-invasive surrogate for central venous pressure [106,107,108,111,115], a sensitive marker of decongestion [104,108,112], and, most importantly, a robust, independent predictor of rehospitalization and death [110,113,114,116,219]. It tells us not just what is happening, but what it means for the future. The clinical application of this is powerful. Is the patient adequately decongested? A falling liver stiffness suggests yes. Is a patient being discharged still at high risk? An elevated stiffness warns of impending readmission. Is a patient with advanced HF a candidate for a ventricular assist device? Pre-operative stiffness provides crucial risk stratification [107,108,111,117,118]. Furthermore, SWE can differentiate phenotypes, showing greater stiffness in the more advanced haemodynamic derangement of heart failure with reduced ejection fraction compared to mid-range EF [120]. The true genius, however, lies not in using these tools in isolation but in weaving their narratives together. This integrated diagnostic approach, moving from conventional ultrasound to CEUS to SWE, forms a logical, stepwise algorithm (Table 5). We start with the broad scan (conventional ultrasound) to suspect congestion. We then zoom in with the functional lens (CEUS) to confirm and localize the microvascular dysfunction. Finally, we apply the structural lens (SWE) to ask the critical question: is this acute edema or chronic scar? The synthesized plot they reveal is transformative. Consider a patient with abnormal liver function: a prolonged hepatic vein transit time on CEUS (functional impairment) coupled with high stiffness on SWE (structural change) suggests acute-on-chronic congestion. After diuresis, if the transit time normalizes but the stiffness remains high, the plot twist is revealed—the backdrop has permanently hardened into cardiac cirrhosis. This changes everything: our prognosis, our therapeutic expectations, our long-term plan. In conclusion, the advent of CEUS and SWE offers a paradigm shift from managing a cardiac pump failure to stewarding a multisystem illness. They provide the chapters previously missing from our clinical assessment, allowing us to read the systemic story of heart failure in vivid detail. By integrating these functional and structural insights, we move beyond reactive guesswork towards a personalized, physiologically grounded, and truly holistic management of one of medicine’s most complex syndromes.

## 5. Conclusions

The incorporation of CEUS and SWE into the diagnostic evaluation of heart failure may significantly enhance the clinician’s ability to detect, quantify, and interpret systemic congestion. While conventional ultrasound remains indispensable for macrovascular hemodynamic assessment, advanced ultrasound modalities add critical insight into microvascular flow and tissue mechanics. Their integration allows for more precise differentiation between reversible and irreversible organ injury, improved guidance of decongestive therapy, and more accurate risk stratification. By embracing this multimodal approach, heart failure assessment evolves from a predominantly cardiac-focused framework to a truly multisystem evaluation, better capturing the diverse and interconnected pathophysiology that characterizes the disease. Prospective studies validating integrated CEUS-SWE protocols against hard clinical endpoints are needed to move this promising diagnostic framework into routine practice and guide personalized decongestion strategies.

## Figures and Tables

**Table 1:** The relationship between invasively measured central venous pressure (CVP) and liver stiffness measurements (LSMs) is examined. In this context, ARFI stands for acoustic radiation force impulse, CVP refers to central venous pressure, HF indicates heart failure, kPa represents kilopascals, LSM denotes liver stiffness measurements, LVAD stands for left ventricular assist device, and m/s means meters per second. The data are reported as either the mean ± standard deviation or median with interquartile range.

Study	Modality	Sample Size (n)	Study Population	LSM	Measured CVP (mmHg)	Correlation (*r*)	*p* Value
Nishi et al. [107]	FibroScan	30	LVAD recipients	13.3 ± 13.0 kPa	8.8 ± 6.9	0.515	<0.01
Potthoff et al. [108]	ARFI	28	LVAD recipients	2.50 ± 0.92 m/s	14.0 ± 6.0	0.793	0.001
Yoshitani et al. [106]	ARFI	38	Decompensated HF	2.03 ± 0.91 m/s	11.8 ± 5.4	0.636	0.014
Taniguchi et al. [110]	FibroScan	31	Decompensated HF	8.5 (5.3–12.0) kPa	9.0 (5.0–12.0)	0.95	<0.001
Kashiyama et al. [111]	FibroScan	55	LVAD recipients	12.7 ± 13.1 kPa	7.4 ± 5.0	0.52	<0.01

**Table 2:** Changes in liver stiffness measurements after intervention. ARFI, acoustic radiation force impulse; kPa, kilopascals; LSM, liver stiffness measurements; LVAD, left ventricular assist device; m/s, meters per second. The sample sizes may differ from Table A2 (in Appendix A) due to variability in obtaining post-intervention LSM. bValues are expressed as mean ± standard deviation or median (interquartile range).

Study	Modality	Intervention	Sample Size (n)	LSM Before Intervention	LSM After Intervention	*p* Value
Millonig et al. [102]	FibroScan	Diuresis	10	40.7 (6.1–51.3) kPa	17.8 (3.3–33.2 kPa)	0.004
Colli et al. [103]	FibroScan	Diuresis	27	8.80 (5.92–11.90) kPa	7.20 (5.2–11.30) kPa	0.003
Hopper et al. [104]	FibroScan	Diuresis	8	11.2 (6.7–14.3) kPa	9.5 (7.3–21.6) kPa	>0.09
Alegre et al. [112]	FibroScan	Diuresis	9	14.7 (8.3–18.8) kPa	8.2 (5.1–11.2) kPa	0.008
Soloveva et al. [113]	FibroScan	Diuresis	149	12.2 (6.3–23.6) kPa	8.7 (5.9–14.4) kPa	<0.001
Yoshitani et al. [106]	ARFI	Diuresis	14	2.37 ± 1.09 m/s	1.27 ± 0.33 m/s	<0.001
Potthoff et al. [108]	ARFI	LVAD placement	23	1.88 (0.92–3.72) m/s	1.43 (0.93–3.67) m/s	<0.001

**Table 3:** Summary of LSM as an independent risk factor for adverse events. ARFI, acoustic radiation force impulse; CV, cardiovascular disease; HF, heart failure; kPa, kilopascals; LSM, liver stiffness measurements; m/s, meters per second; SBP, systolic blood pressure.

Study	Modality	Sample Size (n)	Cut-Off for High LSM Group	Composite Endpoints	Number of Events (n)	Mean Follow-Up Time (Days)	Independent Risk Factor(s) for Event
Saito et al. [116]	FibroScan	105	≥8.8 kPa (on admission)	Death from CV disease, readmission for HF	42	253	LSM
Omote et al. [114]	ARFI	70	≥1.50 m/s (on admission)	All-cause death, worsening HF	26	272	SBP-LSM
Taniguchi et al. [115]	FibroScan	171	>6.9 kPa (on discharge)	Cardiac death, readmission for HF	41	203	LSM
Soloveva et al. [113].	FibroScan	149	>13 kPa (on admission) ≥5 kPa (on discharge)	All-cause death, heart transplant, HF readmission	71	289	LSM at discharge

**Table 4:** Comparison of conventional ultrasound with contrast-enhanced ultrasound (CEUS) and shear wave elastography (SWE). Conventional ultrasound offers a macroscopic view of venous flow and cardiac function. CEUS adds quantitative microvascular assessment, while SWE quantifies tissue stiffness, distinguishing acute congestion from chronic fibrosis. Together, these modalities create a comprehensive evaluation of systemic congestion in heart failure.

Method	What It Measures	Strengths
Conventional US (B-mode, Doppler, VExUS)	Macrovascular flow; Doppler patterns; IVC size; morphology	Widely available; strong physiologic links to venous pressure
CEUS (Functional Pillar)	Microvascular perfusion; transit times; perfusion kinetics	Quantitative transit times; early detection of congestion
SWE (Structural Pillar)	Tissue stiffness; edema vs. fibrosis; structural remodeling	Distinguishes acute vs. chronic damage; strong prognostic value
Combined Use (Integrated Approach)	Hemodynamics + perfusion + tissue response	Comprehensive assessment of systemic congestion

**Table 5:** Flowchart about the integrated use of conventional and advanced ultrasound in heart failure evaluation.

Step	Action	Key Metrics/Findings	Interpretation	Outcome
**1. Initial Screening with Conventional Ultrasound**	Perform bedside B-mode/Doppler ultrasound (e.g., VExUS: IVC, hepatic, portal, renal venous Doppler).	**Findings Suggestive of Congestion:**• Dilated IVC• Plethoric/monophasic hepatic veins• Pulsatile portal flow• Discontinuous intrarenal venous flow	If **negative or inconclusive** → low probability of significant hemodynamic congestion. If **positive or ambiguous** → proceed to CEUS.	Rule out or escalate to Step 2.
**2. Functional and Microvascular Interrogation with CEUS**	Perform targeted CEUS of liver, kidney, spleen (guided by clinical suspicion or US findings).	**Quantitative perfusion metrics:**• Hepatic Vein Transit Time• Renal Cortical Time-to-Peak• Splenic perfusion indices	Prolonged transit times or abnormal perfusion curves indicate microvascular congestion and provide a quantitative baseline.	Confirms presence and severity of hemodynamic congestion; proceed to Step 3 for structural assessment.
**3. Structural and Tissue Characterization with SWE**	Perform SWE of same organs studied with CEUS.	**Tissue stiffness:** measured in kPa or m/s	**Acute/reversible pattern:** stiffness elevated but normalizes after decongestion (CEUS also normalizes). **Chronic/irreversible pattern:** stiffness remains elevated despite normal CEUS (suggests fibrosis).	Distinguishes reversible hemodynamic stress from irreversible organ damage; informs prognosis and therapy intensity.

## Data Availability

The data presented in this study are available on request from the corresponding author due to privacy.

## Appendix A

**Table A1:** Contrast-Enhanced Ultrasound (CEUS)—Principles, Indications, and Advantages [42].

1. Principles	2. Indications	3. Advantages
CEUS is a specialized imaging technique that utilizes intravenous microbubble-based contrast agents to visualize blood flow in real-time The Contrast Agent: Ultrasound Contrast Agents (UCAs) are biocompatible microbubbles (1–10 µm in diameter) consisting of a gas core (e.g., sulphur hexafluoride) stabilized by a phospholipid or albumin shell. Their key property is that they are pure intravascular agents, remaining entirely within the blood pool after injection, unlike CT or MRI contrast which diffuses into the interstitium. Mechanism of Action: When insonated with a specific, low-energy ultrasound beam (Low Mechanical Index—MI), these microbubbles resonate and scatter ultrasound waves non-linearly. Modern ultrasound systems are designed to detect these unique non-linear signals while suppressing the linear signals from surrounding tissues. This results in a pure visualization of the vascular architecture and perfusion without background tissue interference. Real-Time Dynamic Assessment: The core principle of CEUS is the real-time observation of the microbubbles as they flow into (wash-in) and out of (wash-out) a region of interest. This allows for the dynamic assessment of hemodynamics over several minutes. Vascular Phases (Liver Example): In the liver, this dynamic flow provides distinct vascular phase information: ○ Arterial Phase (AP): 10–20 s to 30–45 s post-injection. ○ Portal Venous Phase (PVP): 30–45s to ~120 s. ○ Late Phase (LP): From ~120 s up to 4–6 min. Lesion characterization is based on its enhancement pattern (hyper-, iso-, or hypo-enhancing) relative to the surrounding liver parenchyma during each of these phases. The presence and timing of wash-out (loss of enhancement) in a lesion is a critical differentiator between benign and malignant entities.	The paper outlines a wide and growing range of clinical applications for CEUS across multiple organ systems and clinical scenarios. Focal Liver Lesion Characterization: This is the primary and most established indication. ○ Differentiating benign (e.g., hemangioma, focal nodular hyperplasia) from malignant (e.g., hepatocellular carcinoma (HCC), metastases) lesions in a non-cirrhotic liver. ○ LI-RADS Classification: Characterizing nodules in a cirrhotic liver for the non-invasive diagnosis of HCC according to the standardized CEUS LI-RADS algorithm. Oncology Treatment Monitoring (DCE-US): Using quantitative Time-Intensity Curve (TIC) analysis, either via bolus injection or continuous infusion, to assess changes in tumor vascularization and perfusion in response to anti-angiogenic therapy. Guidance for Interventions: ○ Biopsy: To target the viable, enhancing portion of a tumor and avoid necrotic or cystic areas, significantly increasing diagnostic yield. ○ Ablation Therapy (e.g., RFA): For pre-procedural planning, intra-procedural guidance, and immediate post-procedural assessment to confirm complete treatment and detect any residual viable tumor. Non-Hepatic Applications: ○ Renal: Characterization of complex cysts and solid renal masses. ○ Pancreas: Evaluation of cystic lesions (e.g., IPMN) and solid masses. ○ Inflammatory Bowel Disease: Assessing bowel wall vascularity for activity. ○ Macrovascular: Assessing vessel patency, stenoses, and endoleaks after endovascular repair. Extravascular (Intracavitary) CEUS (EV-CEUS): Using diluted UCA injected into body cavities for applications such as: ○ Vesicoureteral reflux (VUR) in children. ○ Fallopian tube patency (Hysterosalpingo-Sonography). ○ Percutaneous nephrostomy and abscess drainage tract confirmation. ○ Fistula and sinus tract evaluation. Pediatric Imaging: A highly valuable tool due to the lack of ionizing radiation, approved for liver lesion characterization and VUR sonography.	CEUS offers several distinct advantages over other cross-sectional imaging modalities, as emphasized throughout the paper. Real-Time Dynamic Assessment: CEUS provides a continuous, real-time view of blood flow dynamics, capturing the exact timing and pattern of enhancement and wash-out. This is a unique capability not possible with single-time-point CT or MRI. Excellent Safety Profile: ○ No Nephrotoxicity: UCAs are not renally excreted. There is no need to check renal function prior to administration. ○ Low Allergenic Potential: The incidence of severe anaphylactic reactions is extremely low (approximately 0.001%), significantly lower than with iodinated CT contrast agents and comparable to MR agents. ○ No Ionizing Radiation: Eliminates radiation exposure for both patients and operators. Point-of-Care & Repeatability: The examination can be performed at the bedside. It allows for multiple, sequential injections during the same session to evaluate different lesions or to reassess a finding without concern for cumulative dose or radiation limits. Cost-Effectiveness: Generally lower cost per examination compared to CT and MRI, making it particularly valuable in resource-limited settings. Superior Contrast Resolution: The pure intravascular nature of the agent and the high contrast-to-tissue ratio allow for exquisite sensitivity to capillary-level flow, often detecting perfusion differences invisible on other modalities. Ideal for Patients with Contraindications: The go-to alternative for patients with contraindications to iodinated CT contrast (e.g., severe allergy, renal failure) or gadolinium-based MR agents.

**Table A2:** Liver Elastography—Principles, Indications, and Advantages (Based on WFUMB Guidelines) [46].

1. Principles	2. Indications	3. Advantages
The Technique: Elastography is a non-invasive method that assesses tissue stiffness by measuring the speed of shear waves propagating through the liver. Stiffer tissue (from fibrosis) propagates waves faster. Mechanism of Action: Shear waves are generated either by an external mechanical vibration (Vibration-Controlled Transient Elastography—VCTE) or by an acoustic “push pulse” from the ultrasound probe itself (Acoustic Radiation Force Impulse—ARFI techniques, including point and 2D-SWE). The ultrasound system then tracks the wave speed. Quantitative Stiffness Measurement: The result is a quantitative measurement of liver stiffness, expressed in kilopascals (kPa) or meters/second (m/s). Higher values indicate greater stiffness, which correlates with the degree of liver fibrosis. Standardized Protocol: A specific acquisition protocol is recommended to ensure reliability, including patient fasting, proper positioning, and obtaining multiple measurements. The median value is reported, along with an interquartile range/median (IQR/M) as a quality metric (Ferraioli et al., 2018 [79]; Barr et al., 2020 [80]).	Liver Fibrosis Staging: The primary indication is for the non-invasive staging of fibrosis in various chronic liver diseases, including Metabolic dysfunction-associated steatotic liver disease (MASLD), alcohol-related liver disease (ALD), and chronic viral hepatitis B and C (Ferraioli et al., 2024) [46]. Prognostication: Liver Stiffness Measurement (LSM) is used to stratify the risk of future liver-related events (e.g., hepatic decompensation, hepatocellular carcinoma) and mortality. For example, a VCTE LSM < 10 kPa indicates a low 5-year risk (Semmler et al., 2023 [81]). Portal Hypertension Assessment: LSM, often combined with platelet count, is used to rule in or rule out clinically significant portal hypertension (CSPH) and to avoid unnecessary screening endoscopy for varices (e.g., Baveno VI/VII criteria) (de Franchis et al., 2022 [82]). Disease Monitoring: Serial LSM assessments can monitor disease progression or regression in response to treatment (e.g., antiviral therapy, abstinence from alcohol, weight loss) (Semmler et al., 2023) [81]. Pediatric Liver Disease: Useful for diagnosis and follow-up in children with chronic liver diseases like MASLD, biliary atresia, and cystic fibrosis-related liver disease, though specific cut-offs are less defined (Ferraioli et al., 2024) [46].	Non-Invasive Alternative to Biopsy: Provides a reliable, non-invasive method for assessing liver fibrosis, reducing the need for liver biopsy and its associated risks (Ferraioli et al., 2018) [79]. Rapid, Point-of-Care, and Repeatable: The examination is quick (5–10 min), can be performed at the bedside, and allows for easy repetition to monitor changes over time without safety concerns. Excellent Diagnostic Accuracy: Demonstrates high accuracy (AUC >0.85) for diagnosing advanced fibrosis and cirrhosis across various liver disease etiologies (Selvaraj et al., 2021) [83]. Strong Prognostic Value: LSM values are strongly predictive of clinical outcomes, helping to identify high-risk patients who require more intensive management and surveillance. No Ionizing Radiation or Nephrotoxicity: The technique uses ultrasound and is safe for patients with renal impairment, unlike CT or some MRI contrast agents. Cost-Effective: Generally lower cost than MRI-based elastography (MRE) and significantly less than the cost of a liver biopsy, making it accessible for widespread use.

**Table A3:** Table of CEUS Studies for Liver Cirrhosis. This table synthesizes over two decades of research into using Contrast-Enhanced Ultrasound (CEUS) to diagnose liver cirrhosis. The collective findings demonstrate that CEUS is not an anatomical imaging tool, but a functional, hemodynamic one. It works by detecting the accelerated transit of microbubble contrast agents through the liver, a phenomenon caused by the intrahepatic shunts and vascular distortions characteristic of cirrhosis. The data reveals a clear evolution. Early studies using the first-generation agent Levovist (Albrecht 1999, Lim 2005) [84,94] established the core principle, showing dramatically shorter hepatic vein arrival times in cirrhotic patients with near-perfect sensitivity. The subsequent shift to second-generation agents like SonoVue (Lim 2006) [88] refined the technique, leading to larger validation studies (Abbattista 2008, 2016) [92,93] that confirmed high diagnostic accuracy (e.g., 91–100% sensitivity, 93–100% specificity). Crucially, the application of CEUS has expanded beyond simply detecting cirrhosis. Research now focuses on its ability to stage disease severity and quantify portal hypertension, a major complication. Studies like Jeong (2015) [99] and Zocco (2023) [100] show that specific CEUS parameters can non-invasively predict severe portal hypertension with excellent accuracy, rivaling more invasive gold-standard measurements. In summary, the table charts the progression of CEUS from a promising concept to a robust, practical, and highly accurate clinical tool for the comprehensive management of chronic liver disease.

First Author, Year	Patients (Type, Number)	Type of Hepatopathy	Contrast Agent	Sens (%)	Spec (%)	ODA (%)	PPV (%)	NPV (%)	AUC	Advantages	Limits
**LEVOVIST**											
**Albrecht, 1999 [84]**	Adults, 38 (15 Cirr, 12 Non-Cirr, 11 Ctrl)	Mixed Chronic Liver Disease	Levovist	100	100	ND	ND	ND	ND	First pilot study, clear separation of groups.	Small sample size, pilot study.
**Giuseppetti, 2004 [91]**	Adults, 40 (12 Cirr, 16 CLD, 12 Ctrl)	Chronic Liver Disease	Levovist	100	100	ND	ND	ND	ND	Automated, objective measurements.	Small sample size.
**Hirota, 2005 [87]**	Adults, 40 (20 Cirr, 20 Non-Cirr)	Compensated Cirrhosis vs. Non-Cirrhotic	Levovist	ND	ND	Highest among tests	ND	ND	ND	Reflects intrahepatic hemodynamics, high accuracy.	ND
**Lim, 2005 [94]**	Adults, 85 (HCV patients) + 20 Ctrl	Chronic Hepatitis C	Levovist	100 (for Cirr)	80 (for Cirr)	ND	ND	ND	ND	Non-invasive alternative to biopsy, clear disease severity differentiation.	ND
**Lim, 2006 [88]**	Adults, 40 (HCV patients) + 25 Ctrl	Chronic Hepatitis C	Levovist	ND	ND	ND	ND	ND	ND	Direct comparison of two agents.	Shorter HVTT with SonoVue limits disease stage differentiation.
**SONOVUE**											
**Lim, 2006 [88]**	Adults, 40 (HCV patients) + 25 Ctrl	Chronic Hepatitis C	SonoVue	ND	ND	ND	ND	ND	ND	Shorter transit time, similar cardiopulmonary transit time.	Could not differentiate mild from moderate hepatitis.
**Ridolfi, 2007 [95]**	Adults, 52 (16 Cirr, 22 CHC, 14 Ctrl)	Chronic Hepatitis C	SonoVue	ND	ND	ND	ND	ND	ND	Reliable for excluding cirrhosis with portal hypertension.	Not useful for assessing severity of chronic hepatitis.
**Abbattista, 2008 [92]**	Adults, 83 (38 Cirr, 31 CLD, 14 Ctrl)	Chronic Liver Disease	SonoVue	100	93.3	ND	92.6	100	ND	High reproducibility, better than conventional US.	ND
**Li, 2010 [98]**	Adults, 122 (CHB patients)	Chronic Hepatitis B	SonoVue	ND	ND	ND	ND	ND	0.946 (PV-HVTT for S4)	Quantitative, reflects fibrosis severity.	ND
**Berzigotti, 2011 [96]**	Adults, 55 Cirr + Ctrl	Liver Cirrhosis	SonoVue	ND	ND	ND	ND	ND	ND	Novel quantitative tool for hepatic perfusion.	Correlates with liver failure, not a direct diagnostic test for cirrhosis.
**Shi, 2012 [97]**	Adults, 30 (15 Cirr, 15 Ctrl)	Early-Stage Liver Cirrhosis	SonoVue	ND	ND	ND	ND	ND	ND	Useful for quantitative diagnosis of early cirrhosis.	Small sample size.
**Jeong, 2015 [99]**	Adults, 53 Cirr	Liver Cirrhosis (for Portal Hypertension)	SonoVue	92 (for SPH)	89 (for SPH)	ND	ND	ND	0.94 (for SPH)	Useful for estimating severe portal hypertension.	Focus on portal hypertension, not cirrhosis diagnosis per se.
**Abbattista, 2016 [93]**	Adults, 174 (78 Cirr, 82 Non-Cirr, 14 Ctrl)	Chronic Liver Disease	SonoVue	91.1	93.6	ND	ND	ND	0.953	Valid for non-invasive staging, 10-year experience.	ND
**Zocco, 2023 [100]**	Adults, 46 Cirr	Liver Cirrhosis (for Portal Hypertension)	SonoVue	100 (for CSPH)	100 (for CSPH)	ND	ND	ND	1.000 (for CSPH)	Excellent predictor of clinically significant portal hypertension.	Monocentric study.
**Zhou, 2025 [101]**	Adults, 50 Cirr	Liver Cirrhosis (for Portal Hypertension)	SonoVue	ND	ND	ND	ND	ND	0.958 (for HVPG≥10)	Non-invasive prediction of HVPG using spleen parameters.	Focus on portal hypertension, not cirrhosis diagnosis.

**Table A4:** This table provides a systematic overview of the five types of Cardiorenal Syndrome (CRS), a complex condition highlighting the bidirectional interplay between the heart and kidneys. It is organized by CRS type and year of publication to show the evolution of understanding for each category. The table distinguishes between syndromes that are primarily cardio-renal (where a heart problem initiates kidney dysfunction, as in Types 1 and 2) and those that are reno-cardiac (where a kidney problem leads to heart damage, as in Types 3 and 4). Type 5 is unique, as a systemic condition simultaneously affects both organs. Furthermore, it classifies each type as acute or chronic, which is crucial for diagnosis and management. The “Main Relevance” column summarizes the specific contribution of each paper, ranging from foundational pathophysiological reviews (e.g., Ismail, 2012) [154] to modern meta-analyses (Vandenberghe, 2016) [155] and explorations of emerging trends like “Omics” (Neres-Santos, 2023) [159] and SGLT2 inhibitors (Li, 2024) [161]. Collectively, these entries illustrate the progression from defining the syndromes to quantifying their impact and exploring novel diagnostic and therapeutic targets. Collectively, the five CRS subtypes illustrate the intricate and mutually reinforcing relationship between the heart and kidneys. This conceptual framework highlights the necessity for integrated, organ-crosstalk–oriented approaches to diagnosis and treatment, as well as the importance of ongoing research aimed at unraveling the mechanistic pathways that drive these interdependent syndromes.

First Author, Year	CRS Type	Characteristics	Type (Cardio-Renal/Reno-Cardiac)	Acute/Chronic	Prevalence	Incidence	Typical Patients	Main Relevance of Paper
**Ismail, 2012 [154]**	1	Acute cardiac dysfunction (e.g., AHF, ACS) leading to AKI.	Cardio-Renal	Acute	~33% of HF admissions	25% to over 70%	Patients with acute decompensated heart failure or acute coronary syndrome.	Reviews the complex pathophysiology and feedback loops in CRS-1, highlighting its impact on morbidity and mortality.
**Vandenberghe, 2016 [155]**	1	Systematic review of AKI in CRS-1 patients (AHF, ACS, post-cardiac surgery).	Cardio-Renal	Acute	25.4% (by AKI criteria)	25% to over 70%	Patients with AHF, ACS, or post-cardiac surgery.	Meta-analysis quantifying the high occurrence rate and negative impact on mortality and hospital stay in CRS-1.
**Jois, 2012 [156]**	2	Chronic cardiac abnormalities (e.g., CHF) leading to chronic renal dysfunction.	Cardio-Renal	Chronic	Very common comorbidity in CHF	30–60% of patients with chronic heart failure	Patients with chronic heart failure.	Focuses on the importance of recognizing and optimizing heart failure therapies in this chronic syndrome.
**Preeti, 2016 [157]**	2	Chronic heart failure leading to secondary renal impairment.	Cardio-Renal	Chronic	ND	3–15%	Patients with chronic heart failure.	Discusses the significance, unique pathophysiology, and progression of type 2 CRS in the context of the thematic series.
**Chuasuwan, 2012 [158]**	3	Acute kidney injury (AKI) leading to acute cardiac injury/dysfunction.	Reno-Cardiac	Acute	15% to 29%	3–15%	Patients with acute kidney injury (e.g., from ischemia).	Explores the pathophysiology where AKI causes cardiac dysfunction via inflammation and physiological derangements.
**Neres-Santos, 2023 [159]**	3	Acute kidney injury resulting in acute cardiac injury.	Reno-Cardiac	Acute	15% to 29%	ND	Patients experiencing an acute kidney injury event.	Presents future perspectives and challenges in understanding CRS-3, focusing on “Omics” technologies like miRNAs and epigenetics.
**House, 2012 [160]**	4	Chronic kidney disease (CKD) leading to chronic cardiovascular disease.	Reno-Cardiac	Chronic	Very high in CKD, increases with ESRD	76.25% in a population of patients with chronic kidney disease (CKD)	Patients with chronic kidney disease, especially those approaching end-stage renal disease (ESRD).	Reviews the epidemiology, pathophysiology (traditional and non-traditional risk factors), and treatment strategies for CRS-4.
**Li, 2024 [161]**	4	Chronic kidney disease leading to cardiovascular disease.	Reno-Cardiac	Chronic	ND	ND	Patients with chronic kidney disease.	Bibliometric analysis identifying oxidative stress, inflammation, uremic toxins, FGF23-Klotho, and SGLT2 inhibitors as key topics in CRS-4 research.
**Soni, 2012 [162]**	5	Systemic condition (e.g., sepsis, lupus, amyloidosis) causing simultaneous cardiac and renal dysfunction.	Secondary	Both	5–38%	38.4% to 71.4% in critically ill patients	Patients with systemic illnesses like sepsis, systemic lupus erythematosus, or amyloidosis.	Provides an early systematic description and definition of the under-recognized CRS type 5.
**Ilagan, 2022 [163]**	5	Systemic condition causing simultaneous cardiac and renal dysfunction, with a focus on pregnancy.	Secondary	Both	5–38%	38.4% to 71.4% in critically ill patients	Pregnant women with systemic conditions affecting heart and kidneys.	Highlights CRS-5 as an underdiagnosed entity in pregnant women and discusses potential management with serelaxin.
